# Desynchronization Increased in the Synchronized State: Subsets of Neocortical Neurons Become Strongly Anticorrelated during NonREM Sleep

**DOI:** 10.1523/ENEURO.0494-22.2025

**Published:** 2025-03-11

**Authors:** Tangyu Liu, Jeremiah Hartner, Brendon O. Watson

**Affiliations:** Department of Psychiatry, University of Michigan Medical School, Ann Arbor, Michigan 48109

**Keywords:** cross-correlogram, desynchronization, nonREM, SWS, sleep, UP state

## Abstract

We aimed to better understand the dynamics of cortical neurons during nonREM sleep—a state in which neuronal populations are silenced for ∼100 ms of every second due to delta wave fluctuations. This alternation between periods of population spiking (“UP states”) and silence (“DOWN states”) generally synchronizes populations at the 1 s timescale, although some prior work has shown that anticorrelations in nonREM can occur in pairs of neurons that are anticorrelated in wake. We used 24 h recordings of frontal cortical neurons in rats to measure cross-correlation between pairs of neurons in wake, nonREM, and REM. Surprisingly, while most pairs of neurons were synchronized, we found a minority of pairs that showed significant nonREM-induced desynchronization, as indicated by negative cross-correlations in nonREM without equivalent anticorrelation in wake or REM. Interestingly, the degree of anticorrelation within NREM epochs was positively modulated by oscillations in the low-frequency (i.e., “delta” or 1–4 Hz) range, meaning anticorrelation between some pairs increases when correlation increases between other pairs. Furthermore, this effect was mediated by firing during the nonsilent UP state phase of the delta cycle, indicating it is not due to neurons active in the DOWN state. Finally, high-variance spike timing between pairs of neurons and burst spiking during UP states are shown to specifically contribute to the anticorrelation. This state-specific desynchronization during the “synchronized” state represents a new phenomenon that can lead to new understanding of network dynamics during sleep.

## Significance Statement

We know that sleep allows us to maintain health and reinforce memory, and sleep is mostly composed of the “nonREM” state. NonREM includes slow fluctuations in neural activity wherein groups of neurons fire and then halt about once per second, earning it the name the “synchronized state.” Here we report that some pairs of cortical neurons are driven into antisynchrony specifically during nonREM. We find that this is mediated by burst-firing neurons during the active phase of each ∼1 s cycle. This work has implications for a new understanding of how nonREM organizes networks of neurons in the neocortex and will fuel new models of that brain state.

## Introduction

Neuronal spiking mediates functions such as sensory perception, motor control, learning, memory consolidation, and network plasticity. Due to plasticity rules, the timing and coordination of spiking among populations of neurons is believed to determine how networks are reshaped by spike patterns (Buzsáki, 2010), but much remains to be discovered about multineuronal spike patterns in sleep.

A fundamental measure of neural network spiking state is synchrony of spike times across neurons, since multiple spikes are more effective in propagating signals than single spikes. Relatedly, the neocortex is frequently described as being in a “synchronized state” during nonREM sleep (Luczak et al., 2015). This is because during nonREM, prominent, large oscillations have been observed to induce nearly all recorded neurons in neocortical populations within hundreds of millimeters of each other to start and stop firing for hundreds of milliseconds at a time (Luczak et al., 2007; Watson et al., 2016). These oscillations are called delta waves; they occur at 0.5–4 Hz and affect both subthreshold membrane potential and spiking (Steriade et al., 2001). Thus, on a gross level, neurons start and stop firing as groups at 0.5–4 Hz during nonREM inducing gross-scale synchrony. Other delta-rich states, such as drowsiness or inattention, also induce gross-scale synchronization of neurons (Poulet and Petersen, 2008).

These cofiring epochs lasting hundreds of milliseconds between delta waves have been referred to as “packets” (MacLean et al., 2005; Watson et al., 2008; Luczak et al., 2015). Intracellular recordings during these events show simultaneous depolarizations between delta waves, or “UP states,” that lead to firing in most neurons. These alternate with hyperpolarized “DOWN states” that temporally coincide with upward-going delta waves in the electroencephalogram (EEG; Cowan and Wilson, 1994; MacLean et al., 2005). This UP/DOWN dynamic among populations has led to the notion of the synchronized state.

Furthermore, spiking during UP states is not just more frequent than DOWN states but has relatively stereotyped temporal patterning, with certain neurons tending to fire more often or earlier than others (Luczak et al., 2007; Peyrache et al., 2011; Watson et al., 2016). These patterns have led to theories that spiking during UP states may induce plasticity or have homeostatic roles (Levenstein et al., 2016; Liu and Watson, 2020). Consistent with this, later work showed UP state-specific synaptic learning rules in response to neural firing (Bartram et al., 2017; González-Rueda et al., 2018). Furthermore, UP states themselves are coordinated with sharp wave ripples in the hippocampus, which also serve both homeostatic and learning roles (Buzsáki, 2015; Roux et al., 2017; Norimoto et al., 2018). Therefore, much work has focused on correlations driven by these within-UP state timing tendencies.

However, some previous work has shown that synchrony during nonREM or UP/DOWN dynamics is not universal and in fact anticorrelations can be seen during nonREM. First, anticorrelations between neuronal spike trains can be found across many brain states (Euston et al., 2007; Gardner et al., 2019; Okun et al., 2019), including at timescales slower than expected by synaptic inhibition alone (longer than 5 ms; English et al., 2017). Furthermore, anticorrelations at the hundreds of millisecond timescale have been specifically observed during nonREM, despite the assumed overall synchronization by nonREM (Euston et al., 2007; Gardner et al., 2019). Such hundreds of millisecond timescale anticorrelations have been modeled to be driven by mixed inhibitory–excitatory subpopulations due those subpopulations becoming desynchronized at times (Renart et al., 2010). However, importantly for the work in this report, previous work showing anticorrelation in nonREM has largely focused on the preservation in nonREM of anticorrelations already seen in behaving wake states (Euston et al., 2007; Gardner et al., 2019). It has been assumed that the UP/DOWN dynamics during nonREM would generally drive correlation or might preserve prior dynamics but would not specifically induce novel anticorrelation at frequencies near that of the UP/DOWN fluctuation itself.

Here, focus first on nonREM anticorrelations and then map those back to relationships between the same pairs of neurons in other states in 24 h recordings from rat frontal cortex using silicon probes. We report the novel finding that the spiking of many pairs of neurons are actually desynchronized specifically during nonREM, without accompanying anticorrelations in wake or REM states. We then go on to analyze mechanistic contributions to this phenomenon.

## Materials and Methods

### Model simulation

**Figure 1.**
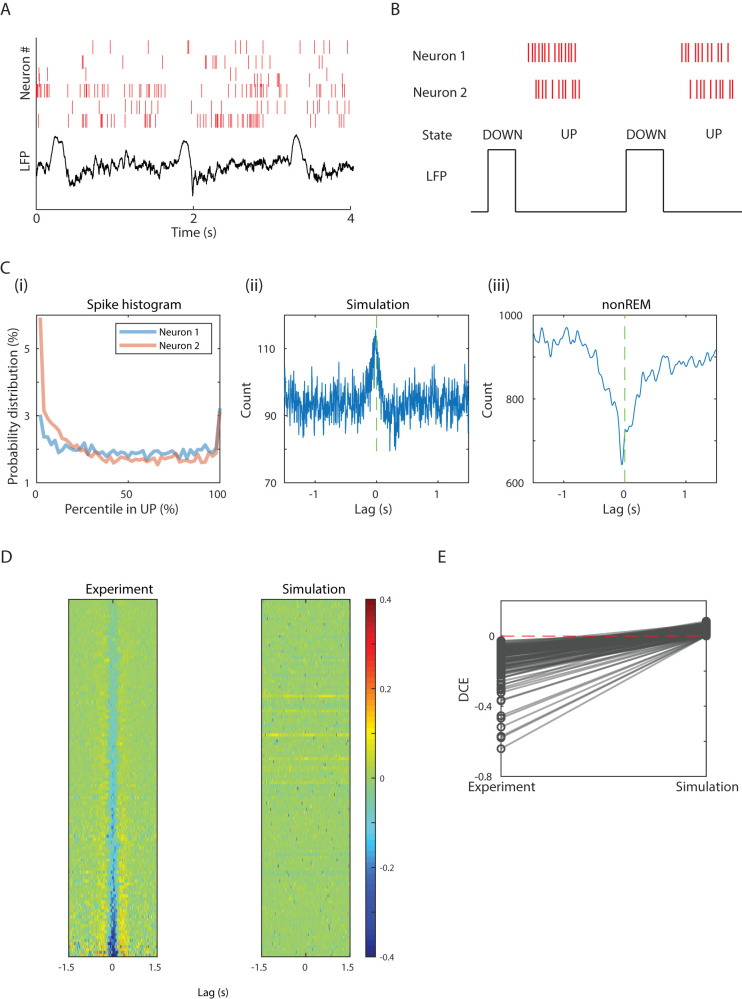
Assumptions of ∼1 Hz synchrony in nonREM are contradicted in some pairs of neurons. ***A***, Example of data from rat cortical multineuronal spiking during nonREM sleep (top) and the corresponding LFP activity (bottom). Note the prominent LFP delta waves (upgoing) that temporally correspond with periods of relative spiking silence. ***B***, Schematic of neuronal spiking activities during DOWN and UP states (note that LFP fluctuations are inverted relative to the membrane potential for which UP and DOWN states are named). ***C***, Traditional lag analysis does not capture the dynamics needed to predict true cross-correlations between pairs of neurons in nonREM sleep. ***i***, The spike count histograms of two neurons collected from all the UP states in a recording. Shown is a probability distribution of spikes at different phases of the UP state ranging from 0% (start of UP state) to 100% (end of UP state). This data was used to seed our simulation. ***ii***, Cross-correlogram (CCG) calculated from two simulated UP state spike trains based on the distribution of the spike count histogram from the neuron 1 and 2 shown in ***i***. This simulation predicts a positive correlation of spiking between pairs of neurons since neurons tend to fire more in UP states than DOWN states. ***iii***, The actual CCG for the neuron 1 and 2 shown in ***i*** and ***ii***. Note that the true CCG from nonsimulated spike trains shows a trough at zero lag, indicating anticorrelation rather than positive correlation. ***D***, Left, All the CCGs with a statistically significant trough from the entire dataset are stacked vertically and sorted by central trough amplitude from baseline (measured by “difference between center and edge” (DCE—see Materials and Methods). Color denotes amplitude of CCG. Right, The corresponding simulations are shown with the same neuron pairs in each row of both plots. ***E***, Comparison of DCE (see Materials and Methods) between experiment and simulation among these pairs with significant troughs in the experiment. All the DCEs in simulation are non-negative.

Two new spike trains are modeled and cross-correlogram (CCG) calculated. The structure of the firing pattern is consecutive UP and DOWN epochs with epoch lengths drawn from one selected recording observed from two neurons. The corresponding firing rates during DOWN states are zero and the firing rates during UP states are based on the spike count histograms collected from the two neurons in the real data shown in Figure 1*Ci*. The spiking pattern distribution was calculated as follows: Each UP state is divided into 50 bins, and spikes are grouped into the bins. All the spike counts in each UP state are converted into probability distribution and then averaged to see the general UP state spike firing pattern. The corresponding CCG count is lower for simulation compared with the real data since we only extracted UP state spikes (rather than all nonREM spikes) from the real data and maintained the same firing rate.

### CCG calculation, normalization, and “difference between the center and the edges” (DCE) calculation

**Figure 2.**
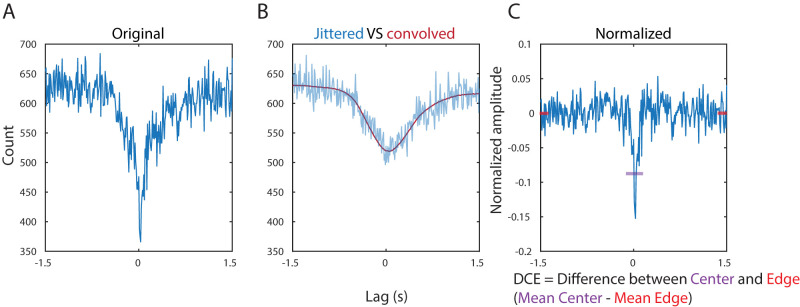
CCG normalization and DCE calculation. ***A***, An example original CCG. ***B***, Averaged CCG with shuffled spike trains (3 times) using 0.5 s jittered window (blue) and the CCG after convolution (1 s triangular window). ***C***, The normalized CCG is defined as log(Original CCG/Convoluted CCG). Illustration of DCE calculation is also demonstrated here.

To calculate the CCG for a specific sleep state, CCGs were first calculated in each individual sleep state epoch (continuous period in that state without breaks) and then all the CCGs were summed up. To compare CCG with various brain states and recordings, CCG normalization is necessary. We first divided the original CCG by another CCG that was calculated from the same spike trains that were locally jittered (Fig. 2). The local jittering eliminated the finer spike timing content in the CCG, and when we divided the original CCG by the jittered one, the coarser spike timing content was eliminated. It was shown that convolving the CCG with a triangular window is equivalent to local jittering but is much more computationally efficient (Stark and Abeles, 2009). We convolved the CCG with a triangular window that was 1 s wide; therefore the divided CCG only contained spike timing content that was finer than 500 ms. This division step is crucial especially for CCG UP state calculation since the duration of UP state epoch can be quite short, rendering bell-shaped CCG before division. The last step of the normalization is to take the logarithm of the divided CCG. The rationale is as follows: Firstly, the logarithm would bring the sides of the normalized CCG to zero. Secondly, we wanted to quantify the degree of correlation and anticorrelation in a comparable level. While the amplitude of the positive correlation in a CCG can go up to an extremely large number, the amplitude of the negative correlation can only go to zero. Taking the logarithm can balance this asymmetry, and we can calculate DCE simply by taking the difference between the center amplitude and the edge amplitude. The center amplitude was calculated from the averaged amplitude in the middle 250 ms window, and the edge amplitude was calculated from the averaged amplitude on both ends (125 ms windows). DCE can be considered as statistically significant if *t* test between center window and edge windows returns significance with an alpha of 10^−4^. For UP state-only CCGs’ DCE calculations, due to their bell curve shaped CCGs that cause undershooting in the convoluted CCGs at the edges, the boundaries are changed to be at the 1 and 2 s lags. Scrambled UP state-only CCGs are calculated by shifting the UP state spike patterns from one of the paired neurons to its previous UP state. The durations of the updated UP states are trimmed to the lesser of the two UP states’ durations. To compare the CCGs in UP states and scrambled UP states, the durations of each UP state are matched. All the nonzero DCE values shown in this paper are statistically significant.

### Electrophysiologic recordings

All animal surgical and recording procedures were in accordance with Institutional Animal Care and Use Committee. Rats (*n* = 6, male) were implanted with silicon probes from NeuroNexus in the prefrontal cortex. Five rats were Long–Evans and one was Sprague Dawley (Table 1, codename “J3_180421'”). All Long–Evans rats used here (Table 1) were datasets from Watson 2016 (available online at https://crcns.org/data-sets/fcx/fcx-1/about-fcx-1).

**Table 1.** Experimental data description

Rat's codename	Recording duration (h)	# of units	# of excitatory units	# of inhibitory units	# of pairs	# of significantly correlated pairs	# of significantly anticorrelated pairs	# of nonREM epochs	# of wake epochs	# of REM epochs
‘BWRat20_101513'	31.9	59	54	5	1,711	56	7	303	385	133
‘Bogey_012915'	29.8	64	61	3	2,016	133	46	321	415	158
‘Dino_080114'	30.6	71	62	9	2,485	226	24	314	390	124
‘J3_180421'	24.4	101	71	30	5,050	185	98	391	443	122
‘Splinter_021015'	23.6	55	13	42	1,485	199	122	327	388	167
‘c3po_160208'	24.0	57	44	13	1,596	328	70	327	365	136

Recordings were confined to the homecage of the animal and the animal was not perturbed or given stimuli. Voltage signals were obtained at 20 kHz using Intan RHD2000 amplifiers with 64-channel headstages. Recordings were stopped and started each 2 h period and were post hoc concatenated over each 24 h and were low-pass filtered to create LFP and were spike sorted with a mixture of KlustaKwik (Long–Evans) and KiloSort2 (Sprague Dawley). LFPs were used to designate sleep states of wake, nonREM, REM using SleepScoreMaster.m for automated prescoring and then TheStateEditor.m for manual checking (both from Watson 2016 and the Buzcode repository: https://github.com/buzsakilab/buzcode).

All locations were verified using post hoc histological staining after lesions of probe tip electrodes using stimulation at 5 μV for 5 s.

### Stacked CCG

**Figure 3.**
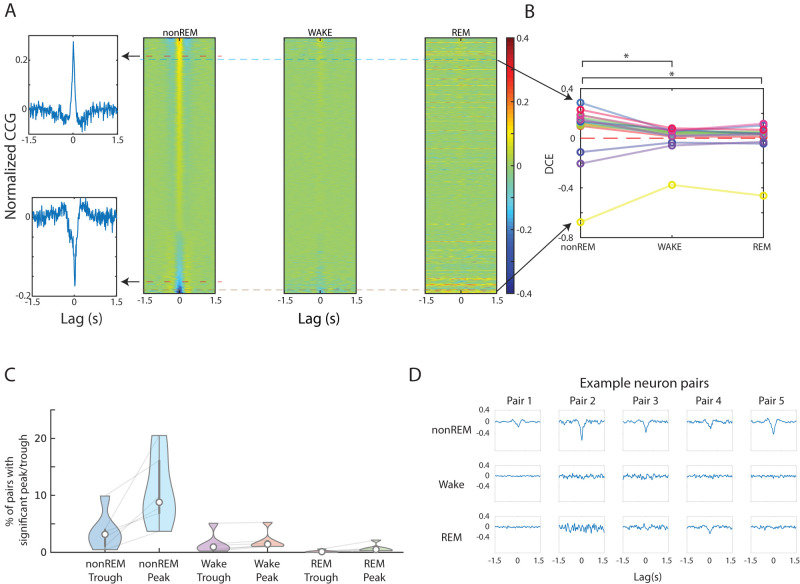
NonREM sleep shows higher amplitude hundreds of millisecond timescale cross-correlations in both positive and negative directions. ***A***, Stacked CCGs with statistically significantly nonzero DCE (either positive or negative) from the full dataset. Left, Top is an example CCG from a pair of neurons showing a peaky (positive correlation) CCG in nonREM; bottom is a CCG from a pair with CCG dominated by a trough (anticorrelation). Right, Stacked CCGs in wake and REM states are sorted by the same DCE in nonREM ranking (horizontal lines continue to represent the same pairs) but show lower amplitude peak/trough for the same pair of neurons. Extended Data Figure 3-1 shows a similar display but including all pairs from this recording (not only those with significant peak/trough). ***B***, DCE variation with the same pairs over states. Only pairs with nonzero DCEs in all three states are included. The mean of the absolute value of DCE in nonREM is significantly larger than wake (*p* = 0.005) and REM (*p* = 0.009), but DCE in wake is not larger than REM (*p* = 0.97; Tukey–Kramer). ***C***, Quantifying state-wise effects via proportion of pairs showing significant correlation patterns. The statistics of trough and peak percentage of all neuron pairs averaged across all our animals. Gray lines indicate connections between percentage values for individual recordings. ***D***, Example neuron pairs demonstrating greater troughs in nonREM. Extended Data Figure 3-2 shows stacked CCGs sorted by different sleep states: same data as 3–1, but sorting order is changed. Extended Data Figure 3-3 shows cross-state comparisons of CCGs of neurons shown in one recording. Extended Data Figure 3-4 shows CCGs of neurons with significantly nonzero DCEs in both wake and nonREM in one recording. Extended Data Figure 3-5 shows downsampled versions of all pairs in the recording to compensate for spike rate differences. Our observation of peaks and troughs in nonREM being stronger remains, indicating lack of evidence for a differential sampling effect.

All the CCGs in a recording are stacked together, and they are sorted based on the DCE value calculated. In Figures 3 and 5, all the stackings are sorted based on the DCE value calculated during nonREM state.

### CCG downsampling

To avoid sampling issues when comparing CCGs in different sleep states, we downsampled spikes to match the state with the least spikes. First, we sampled random intervals without replacement in nonREM and wake to match the same total epoch duration of REM state, since REM has the least total duration. Next, the spikes in each state were downsampled to match the states with the least spikes. This process was iterated three times in our simulation, and the DCEs were calculated and averaged.

### Burst spikes removal and random spikes removal

Log ISI histograms were calculated for each neuron. A Gaussian mixture model was used to separate the bimodal distribution. Only neurons with two peaks that are 1 log_10_(s) apart were selected. The burst threshold was set at two standard deviations toward the separatrix from the mean of the faster timescale Gaussian curve. Once the threshold was set, any three consecutive spikes (or more) that had ISIs less than the threshold were removed. Based on the number of burst spikes removed, the same amounts of random spikes during nonREM state were removed for random spikes removal scenario. Burst spikes were also removed under certain analyses by moving the burst threshold using other principles (i.e., matching spike removed by other methods for comparison) or removing only a percentage of the burst spikes after the threshold was set as described above.

### DCE relative to real DCE

To compare the effect of spikes removal from pairs to pairs, DCE relative to real DCE is defined as follows:

$$\frac{\mathrm{Post}\;\mathrm{removeal}\;\mathrm{DCE}}{\mathrm{Original}\;\mathrm{DCE}}\{\begin{array}{c} >1,\;\mathrm{if}\;\mathrm{the}\;\mathrm{effect}\;\mathrm{of}\;\mathrm{correlation}\;\mathrm{or}\;\mathrm{anticorrelation}\;\mathrm{is}\;\mathrm{amplified}\\ \mathrm{between}\;0\;\;\mathrm{and}\;\;1,\;\mathrm{if}\;\mathrm{the}\;\mathrm{effect}\;\mathrm{of}\;\mathrm{correlation}\;\mathrm{or}\;\mathrm{anticorrelation}\;\mathrm{is}\;\;\mathrm{reduced}\;\\ <0,\;\mathrm{if}\;\mathrm{the}\;\mathrm{relation}\;\mathrm{is}\;\mathrm{reversed}\;(\mathrm{corr}\to \;\mathrm{anticorr}\;\mathrm{or}\;\mathrm{anticorr}\to \mathrm{corr}) \end{array}.$$

### Code accessibility

The MATLAB codes for the model and for calculating DCE are available at https://github.com/BrendonWatsonLab/DCE and https://github.com/buzsakilab/buzcode. We ran the codes using a computer with Intel(R) Xeon(R) CPU E5-2640 v3 @ 2.60 GHz and 500GB RAM.

10.1523/ENEURO.0494-22.2025.d1 Extended Data Download Extended Data, ZIP file.

## Results

### nonREM dynamics based on average statistics versus true pairwise spiking timing

We used 24-h-long recordings from the prefrontal cortex of male Sprague Dawley and Long–Evans rats. These were recorded with silicon probes with spike sorting performed either by KlustaKwik or KiloSort (new recordings). See Materials and Methods for details.

Our first step was to determine what cell–cell spike synchronization would be expected from current knowledge of UP state dynamics (Fig. 1*A*,*B*). CCG is a method to measure overall spike timing relationships between pairs of spiking neurons by quantifying tendencies for the occurrences of various lags between pairs of spikes—for each pair of neurons. Synchrony would manifest in a CCG by the presence of a peak of spike–spike incidence at zero lag.

We built a model to test what CCG pattern would be generated by the spiking pattern generated by averaged per-cell spike statistics from in vivo nonREM sleep (Fig. 1*C*). For this, we took data from multiple simultaneously recorded neurons in nonREM and gathered their spiking distributions across UP states, including the spike timing distributions relative to UP state start for each cell (Fig. 1*Ci*). This was the same approach used in previous work to determine UP state spiking properties (Luczak et al., 2007). We repeatedly sampled from these distributions for pairs of neurons to create simulated spiking across multiple simulated UP states. We then generated CCGs from this statistically generated spiking for pairs of neurons across simultaneous UP states. We also normalized the CCGs and quantified the degree of peaks and troughs with a metric called DCE (for details, see Materials and Methods and Fig. 2). This essentially is a subtraction between values near zero lag and those from higher lag baseline periods (see Materials and Methods). This model showed that 26% of the CCGs we generated have positive DCEs (Fig. 1*Cii*), and the rest of them have DCEs not significantly different from 0. None showed anticorrelations or CCG troughs.

However, ∼2% of the CCGs from our animal data during nonREM instead showed trough in the middle (Fig. 1*Ciii*), and ∼8% of them have positive DCEs. For the example neuron pair shown in Figure 1*C*, we simulated the spikes 100 times and find the distribution of DCEs. The true DCE is highly unlikely to be due to noise fluctuations around the mean statistics (*z*-test, *p* < 5 × 10^−324^). This indicated that the specific spiking occurring during individual UP states departs from average statistics in a manner that can create antisynchrony. More fundamentally, these nonREM-specific antisynchronized neurons have not been reported in neocortex during the synchronized state in natural sleep.

We further collected CCG pairs with a statistically significant trough in our recordings and stacked them to visualize the population-wide pattern (Fig. 1*D*, left). The corresponding simulations are also stacked and shown on the right. The troughs are not seen in simulations. In Figure 1*E*, we quantify the effects shown in 1*D* using DCE.

### Statistics of synchrony versus antisynchrony

Next, we stacked the CCGs in our dataset to create images of CCGs from multiple pairs of cells—from various brain states (Fig. 3*A*). The stacked CCGs are sorted from deepest trough to highest peak based on CCGs in the nonREM state. Only CCGs with a significant peak or trough are displayed (although we quantified all CCGs and significances for all pairs in all states, and the full CCG stacks are shown in Extended Data Fig. 3-1). We aligned CCGs such that CCGs from the same pair of neurons was displayed at the same vertical location in images for nonREM, wake, and REM sleep. It is clear from this visualization that nonREM shows both greater peaks and troughs in CCGs. It appeared that wake and REM also shared peak/trough tendencies but to a smaller degree (Fig. 3*B*). For all of the nonzero nonREM, DCEs are more positively correlated with wake DCEs (*R* = 0.33, *p* = 3.6 × 10^−77^) and less so with REM DCEs (*R* = 0.16, *p* = 2.2 × 10^−18^).

10.1523/ENEURO.0494-22.2025.f3-1 Figure 3-1 CCG Pairwise comparison between sleep states from all recordings.(A) Pairwise comparison between nonREM and wake on all the pairs in the dataset that have nonzero values in these two states (P = 2.58 × 10^−36^).(B) Pairwise comparison between nonREM and REM on all the pairs in the dataset that have nonzero values in these two states (P = 2.01 × 10^−14^).(C) Pairwise comparison between wake and REM on all the pairs in the dataset that have nonzero values in these two states (P = 0.5).(D) Stacked CCGs from full dataset. Each stack is sorted by its own middle amplitudes. Download Figure 3-1, TIF file.

10.1523/ENEURO.0494-22.2025.f3-2 Figure 3-2 Stacked CCGs between sleep states from all recordings sorted by different sleep states.(A) All the Stacked CCGs are sorted by nonREM.(B) All the Stacked CCGs are sorted by wake.(C) All the Stacked CCGs are sorted by REM. Download Figure 3-2, TIF file.

10.1523/ENEURO.0494-22.2025.f3-3 Figure 3-3 Comparison of CCG samples between states in one recording. CCGs with their DCEs shown in Figure 3B are displayed here. The CCGs are sorted by the DCEs in nonREM. Each column of three CCGs is from one neuron pair. The red dot-lines in the top left panels denote the width of trough in Wake. CCGs were Gaussian smoothed at width 10  ms. Download Figure 3-3, TIF file.

**Figure 4.**
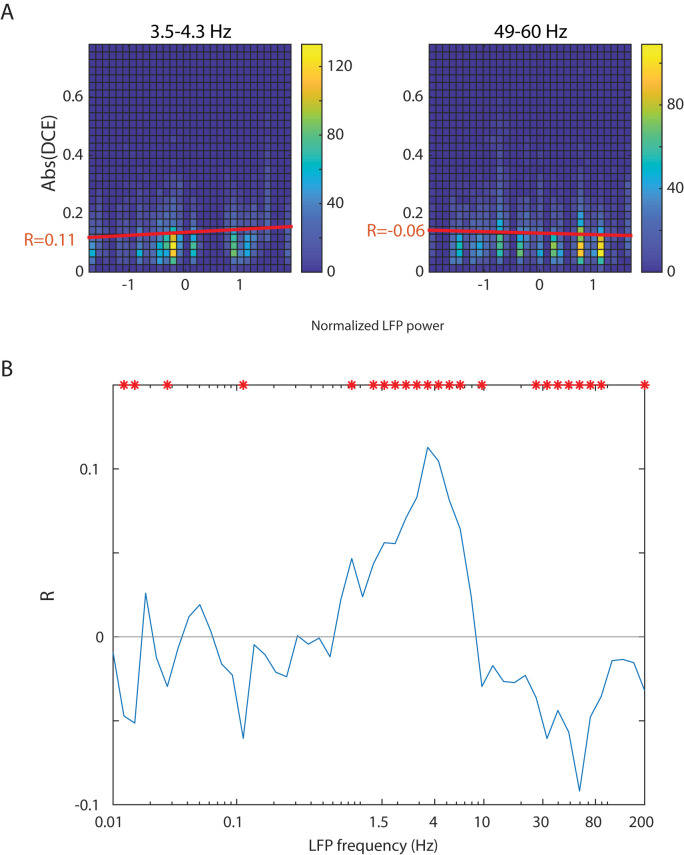
The amplitude of cross-correlations is positively correlated with power of low-frequency oscillations and negatively correlated with power of high-frequency oscillations. ***A***, Relation between the absolute value of DCE and normalized LFP power within nonREM sleep—in two example frequency bands (left and right). Shown are density plots of population DCE versus LFP power in each 3 h of all recordings within our dataset. Density plots are used instead of standard *x*–*y* scatterplots to better show relative densities of many very dense points. The recordings are divided into epochs of 3 h. DCEs are calculated for all pairs with negative DCE during nonREM found within each 3 h epoch. The corresponding LFP power during those epochs are calculated and they are normalized over the time axis within each recording, to compare between recordings. The examples shown are data for the 3.5–4.3 Hz (left) and 49–60 Hz (right) frequency bands. At 3.5–4.3 Hz, the Spearman correlation coefficient *R* = 0.11 (*p* = 7.1 × 10^−15^), and at 49–60 Hz *R* = −0.06 (*p* = 9.6 × 10^−5^). See red lines of best linear fit. Full spectrum density plots are shown in Extended Data Figure 4-2. ***B***, Relation between the *R* value calculated based on the process shown in ***A*** for each of many frequency bands. Significant *R* values are denoted with red asterisks (*p* < 0.05).

10.1523/ENEURO.0494-22.2025.f3-4 Figure 3-4 Comparison of CCG between nonREM and Wake in example pairs. All the pairs with nonzero DCE in both nonREM and Wake in one recording are compared. Each column of two CCGs is from one neuron pair. The CCGs are sorted by the DCEs in nonREM. CCGs were Gaussian smoothed at width 10  ms. Download Figure 3-4, TIF file.

**Figure 5.**
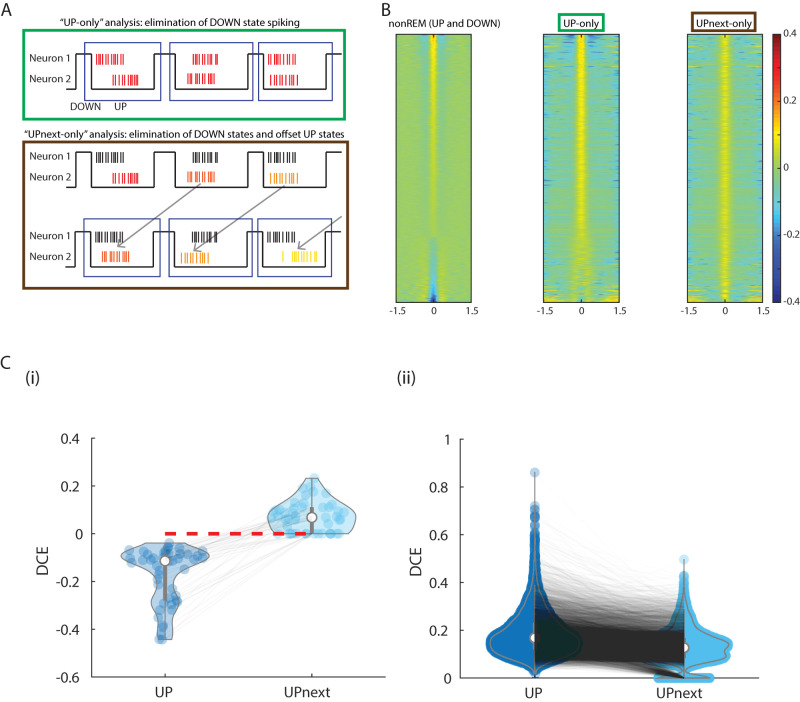
Spiking details across neurons within each UP state play a role in forming anticorrelations. ***A***, Diagrams of testing methods to determine the role of UP state spikes in nonREM cross-correlations. Top, “UP” analysis: CCGs are calculated for each neuron pair within each UP state, but not within DOWN states or any non-UP state epoch of nonREM. CCGs are then summed over all the UP states within a recording. Bottom, “UPnext-only” analysis: CCGs are calculated between the spike train of one neuron from UP state *N* and the spike train of a second neuron from UP state N + 1 (next UP state). Again, CCGs are then summed over all the UP states. As shown in Figure 1*C*, we know that neurons have typical lags in their firing relative to UP states, but we know less about the importance of any inter-UP state variation in that typical lag. These analyses test the importance of the simultaneous dynamics within each UP state across neurons. ***B***, Comparison of the stacked CCGs between different analyses. At left are the raw nonREM CCGs showing both significant peaks (top) and troughs (bottom). The center shows the UP analysis: CCG patterns are similar to nonREM state, despite the exclusion of any spikes during DOWN states or non-UP state times. The UP states here are trimmed to compare with the scrambled UP states (see Materials and Methods). This indicates that UP state spiking dynamics themselves can create both positive and negative CCGs. However, in the “UPnext-only” analysis, the nonREM CCG patterns are destroyed, indicating that details of simultaneous spiking between pairs of neurons in simultaneous UP states are needed for negative cross-correlations. See Extended Data Figure 5-1 for all pairs, rather than only significantly correlated/anticorrelated pairs. ***C***, DCE Pairwise comparison between UP states and the corresponding scrambled UP states (“UPnext-only”). ***i***, Only negative DCEs in UP states are selected. The corresponding DCEs in “UPnext-only” states are non-negative and significantly different (*p* = 1.03 × 10^−21^). ***ii***, Only positive DCEs in UP states are selected. DCEs in “UPnext-only” states are non-negative and significantly smaller (*p* < 5 × 10^−324^) with a mean reduction of 23%.

10.1523/ENEURO.0494-22.2025.f3-5 Figure 3-5 Downsampled CCG Pairwise comparison between sleep states from all recordings.(A) Stacked and downsampled CCGs across sleep/wake states from full dataset to demonstrate the range of correlation structures. Each stack is sorted by nonREM CCGs’ middle amplitudes. All CCGs are shown including zero and nonzero DCE.(B) Comparison between nonREM, wake, and REM on all the pairs in the dataset that have nonzero values in all three states (One-way ANOVA P = 3.17 × 10^−11^; nonREM VS wake P = 1.83 × 10^−9^; nonREM VS REM P = 1.13 × 10^−9^; REM VS wake P = 0.74).(C) Pairwise comparison between nonREM and wake in all the pairs in the dataset that have nonzero values in these two states (P = 6.25 × 10^−12^).(D) Pairwise comparison between nonREM and REM in all the pairs in the dataset that have nonzero values in these two states (P = 7.31 × 10^−19^).(E) Pairwise comparison between wake and REM in all the pairs in the dataset that have nonzero values in these two states (P = 0.1). Download Figure 3-5, TIF file.

We further quantified these observations and found that for the pairs that have nonzero DCE across all states, the mean of the absolute value of DCE (both peaks and troughs) in nonREM is significantly larger than wake (*p* = 0.005) and REM (*p* = 0.009), and there is no significant difference between wake and REM (*p* = 0.97; Tukey–Kramer).

Tested on all of our recordings, as expected from previous understanding of the synchronized state, a greater number of pairs show significant CCG peaks during nonREM (10.8 ± 6.3%; Table 2). However, CCGs with a trough in nonREM were approximately one-third as frequent at 3.6 ± 3.4% (Fig. 3*C*). After testing significance of DCE for each pair for each state, we carried out statistical comparisons with the following findings. For nonzero DCE pairs during nonREM, the mean of the absolute value of DCE is significantly larger than the corresponding wake (*p* = 9.6 × 10^−10^) and REM (*p* = 9.6 × 10^−10^) pairs, but no difference between wake and REM (*p* = 0.07; Tukey–Kramer) pairs. For nonzero DCE pairs during wake, the mean of the absolute value of DCE are not different between wake and the corresponding nonREM pairs (*p* = 0.06), but they are both significantly larger than the corresponding REM pairs (*p* = 9.6 × 10^−10^). For nonzero DCE pairs during REM, the mean of the absolute value of DCE are not different between REM and the corresponding nonREM pairs (*p* = 0.83), but they are both significantly larger than the corresponding wake pairs (*p* = 9.1 × 10^−4^ and *p* = 7 × 10^−3^; Tukey–Kramer).[Table T2]

**Table 2. T2:** The statistics of trough and peak percentage of all neuron pairs averaged across all our animals

Trough %			Peak %		
nonREM	Wake	REM	nonREM	Wake	REM
3.6 ± 3.4	1.5 ± 1.8	0.2 ± 0.2	10.8 ± 6.3	2.0 ± 1.6	0.7 ± 0.7

Furthermore, we find that if a trough is going to be present, it is more likely present in nonREM. For all the 14,343 DCE pairs in our dataset, 2.31% of them have negative DCEs during nonREM but zero DCEs during wake, while 0.66% of them have negative DCEs during wake and zero DCEs during nonREM. Between nonREM and REM, 2.56% of them have negative DCEs during nonREM but zero DCEs during REM, while 0.06% of them have negative DCEs during REM and zero DCEs during nonREM. Between wake and REM, 0.91% of them have negative DCEs during wake but zero DCEs during REM, and 0.04% of them have negative DCEs during REM and zero DCEs during wake. This shows that CCGs with negative DCE during nonREM do not necessarily follow the same patterns as during wake or REM (Fig. 3*D*).

To address sampling issues when comparing CCGs across different states (in particular with REM often having shorter duration and therefore fewer spikes), we downsampled spikes to match spike counts in each state (see Materials and Methods). The results are consistent with what was found without downsampling (Extended Data Fig. 3-2).

### Local field potential correlates of desynchronization

Given the nonREM state dependence of trough occurrence, and given the presence of prominent delta oscillations in the local field potential (LFP) in nonREM, we investigated whether delta power might modulate these even within nonREM sleep.

We first found that in general, a given pair keeps its correlation/anticorrelation tendencies across 24 h cycles (Extended Data Fig. 4-1*A*). We then divided the recordings into epochs of 3 h and calculated DCE within each epoch for all the pairs that showed trough patterns during nonREM. These relatively long 3 h epochs were required to generate sufficient CCGs to have good statistics. We then correlated oscillatory band power versus DCE within 3 h-long periods, but only using the spiking from within nonREM. We quantified the correlations between the absolute value of DCE and the normalized LFP power within corresponding nonREM in the same 3 h time window (*Z*-scored and outliers removed) for different oscillatory frequency bands. For example, Figure 4*A* shows that LFP power and abs(DCE) are positively correlated at 3.5–4.3 Hz frequency band (left, *R* = 0.11, *p* = 7.1 × 10^−15^) but negatively correlated at 49–60 Hz (right, *R* = −0.06, *p* = 9.6 × 10^−5^). Full data for each abs(DCE)-LFP band correlation is shown in Extended Data Figure 4-2*A*, and the resultant *R* values are plotted in Figure 4*B*. We also separated the 3 h epochs with positive DCE from negative DCE and analyzed both without taking the absolute value of the DCEs and found consistent results (Extended Data Fig. 4-2*B,C*). This analysis indicated that the power of delta-band oscillations positively modulates amplitudes of DCE but power of gamma-band oscillations negatively modulates them.

10.1523/ENEURO.0494-22.2025.f4-1 Figure 4-1 Strengths of pairwise correlations across the population are coordinated over time.(A) Recordings are divided into epochs of three hours. The twenty neurons that are the least synchronized with other neurons (neurons that have the most negative DCE counts during nonREM) are selected for this display. Their DCEs during nonREM at those three-hour epochs are then calculated and shown as the matrix elements. Color scale shows DCE value with each value in the matrix representing the DCE of one neuron pair. Note overall similarity of DCE matrix over multiple hours of recording.(B) At left, time courses of DCE for fifty example pairs with the most negative DCEs during nonREM. At right, standard deviations respectively were calculated for each pair and were shown sorted by its DCE value during nonREM epoch.(C) Comparison between the mean std from the original matrix and matrices where values within each three hours are shuffled (Z-test, P = 1.4 × 10^−168^). The std of the real data is much less than the shuffled, showing the conservation of the correlation structure over time.(D) Correlation between pairs over time. As one pair of neurons becomes more strongly correlated, do others at the same time? The amplitude of DCE between pairs tends to be positively correlated from one timepoint to the next.(i) The absolute values of the time courses of DCE for the twenty neurons’ pairs are first taken. The Pearson correlations between the resultant time courses for each pair are then calculated. Only significant correlations are counted (P < 0.05). The absolute values of the correlation coefficient R are taken and the distribution of the negative Rs and positive Rs are shown. Generally, we see many more counts of significant positive correlations than negative correlations.(ii) Similar approach to (i), but without taking the absolute values of the time courses of DCE. Note the lower amplitude red line in (i), showing that most of the negative Rs are contributed by larger positive DCEs coinciding with smaller negative DCEs. Download Figure 4-1, TIF file.

10.1523/ENEURO.0494-22.2025.f4-2 Figure 4-2 Full data for Figure 4. Separating three-hour epochs with positive DCE from negative DCE and analyze them independently yields consistent result(A) Density plots of mean LFP power versus the absolute values of DCE in each frequency band shown in Figure 4. For density plots with statistically significant R (P < 0.05), the best linear fits are drawn as red lines.(B) Grouping three-hour epochs with positive DCE and find the R values of mean LFP power versus DCE for each of many frequency bands. Relation between the R values and frequency bands are shown. Significant R values are denoted with red asterisks (P < 0.05).(C) Similar approach to (B) but for epochs with negative DCE. Download Figure 4-2, TIF file.

10.1523/ENEURO.0494-22.2025.f4-3 Figure 4-3 Alternative analysis to verify the correlation between band power and DCE in nonREM states.(A) Pearson Correlation between the LFP power and the DCEs calculated in an alternative way. Given a frequency band, each nonREM epoch is ranked into one of four power quartiles and the CCGs are generated for each pair. Correlation between the power and the DCEs calculated from the CCGs in that frequency band can then be calculated. Significant R values are denoted with red asterisks (P < 0.05). Qualitatively similar overall findings to figure 4B, despite different methodology.(B) Raw data underlying plot in (A). For density plots with statistically significant R (P < 0.05), the best linear fits are drawn in red lines. Download Figure 4-3, TIF file.

10.1523/ENEURO.0494-22.2025.f4-4 Figure 4-4 NonREM epoch duration is positively correlated with delta power and negatively correlated with gamma power.(A) We initially found duration of epoch correlates with delta power and negatively correlates with gamma power (similar to 4-4B, but not shown), but to prevent contamination and overly strong findings by ramp-up and ramp-down dynamics, we analyzed only plateaus of each epoch using the following analysis. We found putative contamination of findings by the fact that regardless of epoch duration,timecourse of delta power (i) in the first 200 secs and (ii) in the last 100secs of each nonREM epoch (>200 secs) did not vary. This leads to short duration epochs having low delta and high gamma values not representative of their plateau value, potentially falsely over-driving correlations. Gamma powers are shown in (iii) and (iv). Each power trace is shown as a grey line. Mean values are shown in red lines and standard deviation are denoted by light red area.(B) After correction: pearson correlation between the duration of nonREM epochs (>200 secs) and the mean LFP power in each frequency band. Band power in the first 120 secs and last 50 secs are removed when averaging. Significant R values are denoted with red asterisks (P < 0.05).(C) Raw data underlying plot in (B). For density plots with statistically significant R (P < 0.05), the best linear fits are drawn in red lines. Download Figure 4-4, TIF file.

A similar analysis that does not rely on use 3 h windows generates similar findings (Extended Data Fig. 4-3). In this analysis, we do not use time bins but rank each nonREM epoch into one of four power quartiles and generate a CCG for each neuron pair in each quartile—this combining into quartiles is needed to generate high quality CCGs not able to achieve with single nonREM epochs. All the DCEs here are negative so taking the absolute value are not necessary. We find that low LFP oscillation frequencies negatively correlate with DCE while high frequencies correlate positively. We then deepened our analysis to better understand these differences in band powers between nonREM epochs. We examined band power traces during nonREM epoch and noticed that the power ramp up and ramp down have similar lengths (time constant) regardless of epoch duration (in delta and gamma bands; Extended Data Fig. 4-4*A*; Trachsel et al., 1988). Next, we examined the relation between epoch lengths and averaged band power strengths. Since in shorter epochs the power ramp up and ramp down would contribute more weight when calculating mean power, we only selected epochs with duration over 200 s and also removed the first 120 s and last 50 s of the band power when taking averages to find the central band power mean value. We found that longer epochs showed more delta power and less gamma power (Extended Data Fig. 4-4*B,C*). This suggests a biological modulator of these frequency bands per nonREM episode—which then in turn modulates DCE.

We thus show here, going beyond the data in Figure 1, that not only does nonREM increase anticorrelations in some pairs, but stronger delta or weaker gamma within nonREM also correlates with increased anticorrelation (and more delta/less gamma occur in longer nonREM episodes).

### Contribution of UP state-specific spiking to anticorrelation

Given that the initial model we generated did not demonstrate anticorrelation, despite being based on the real statistics of spiking of each neuron, we wondered if the specific spike patterns during individual UP states would contribute to the unexpected trough patterns.

First, we examined whether UP versus DOWN state dynamics contributed, given recent reports of DOWN state spiking in some neurons (Valero et al., 2021). To do this, we extracted spiking for each UP state for each cell—excluding DOWN states. Then for each pair of cells simultaneously recorded, we calculated CCG for each single UP state. We then summed the CCGs for all UP states for each pair to determine the overall CCG for that pair from UP states only (no DOWN states; Fig. 5*A*, top). We found that CCGs from the original whole-nonREM were similar to UP state-only CCGs shared similar dynamics (Fig. 5*B*, middle). Negative DCEs in nonREM are positively correlated with DCE values in UP state-only CCGs (*R* = 0.75, *p* = 4.6 × 10^−132^ for untrimmed UP states, and *R* = 0.24, *p* = 3.4 × 10^−11^ for trimmed UP states; see Materials and Methods). This indicates that spiking during DOWN states is not necessary to create the anticorrelations we observed.

10.1523/ENEURO.0494-22.2025.f5-1 Figure 5-1 Complete comparison of the stacked CCGs in nonREM, UP-only, and “UPnext-only” states from all dataset (not restricted to significant pairs). Each stack is sorted by nonREM CCGs’ middle amplitudes. All CCGs are shown including zero and nonzero DCE. Results are consistent with significant-only pairs. Download Figure 5-1, TIF file.

We wondered whether the specific spiking dynamics between a pair of neurons during each individual UP state might create the anticorrelations. To test this, we scrambled the UP state spike patterns by shifting the UP state spike patterns from one of the paired neurons to its previous UP state (Fig. 5*A*, bottom). After the scrambling, the troughs were lost (Fig. 5*B*, right), and in pairs with negative UP state-only DCEs, the original and scrambled CCGs’ DCE are uncorrelated (*R* = −0.05, *p* = 0.62). This indicated that details of paired dynamics within each UP state create anticorrelations as shown by CCG troughs.

We quantify this effect across all pairs with significant trough CCGs in the full dataset (Fig. 5*C*). We find that all of the scrambled UP states (“UPnext-only”) are non-negative, regardless of the values of the DCEs in their original UP states. Negative DCEs in UP states are significantly different from DCEs in “UPnext-only” state (*p* = 1.03 × 10^−21^). Additionally, in regard to pairs with peaky CCGs, positive DCEs in UP states are significantly larger than DCEs in “UPnext-only” state (*p* < 5 × 10^−324^), with a mean reduction of 23%.

### UP state spike timing differences are more prominent in anticorrelated pairs

**Figure 6.**
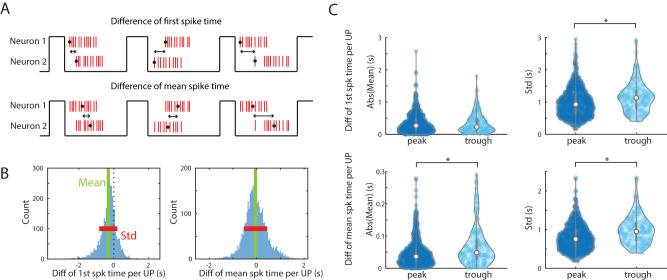
Dissecting within-UP state interactions that influence anticorrelation: Role of differences in in UP state-based spiking lag between pairs of neurons. ***A***, Diagram of comparisons used. Top, Difference of first spike time between pairs of neurons, calculated in each UP state. Bottom, Difference of mean spike time between pairs in each UP state are also calculated. ***B***, Quantification of comparisons used: The differences calculated in ***A*** are collected across all UP states for each pair of neurons. Results for one pair is shown. The mean and the standard deviation (SD) can be calculated for each neuron pair for each metric in ***A***. ***C***, Comparison of the described metrics between all the pairs with negative DCE (trough) and positive DCE (peak) in the full dataset. The SD of difference of first spike time per UP state is higher for pairs in trough than in peak (*p* = 5.8 × 10^−6^). The mean of the difference of mean spike time mean is higher for pairs with troughs (*p* = 1.6 × 10^−9^), as is the SD of the difference of mean spike times higher for pairs in trough (*p* = 9.2 × 10^−13^). Thus, standard deviations of timing differences are consistently higher in trough pairs than peak pairs, indicating that variable timing may lead to toughs.

We next sought to analyze which aspects of UP state spiking dynamics affected the anticorrelation. We began with concepts from previous work (Luczak et al., 2007; Peyrache et al., 2010; Watson et al., 2016), suggesting that each neuron has a typical spike time per UP state. To do this, we used the mean first spike time and mean overall spike time per cell over all UP states. We then quantified the difference in each metric between paired neurons to determine if neurons with more different spike times had greater anticorrelation (Fig. 6*A*). The mean and standard deviation (SD) of that difference for each timing metric are calculated for each pair (Fig. 6*B*). Next, we compared these distributions between all the pairs showing a CCG peak and pairs showing significant correlation (peak in CCG) or anticorrelation (trough in CCG).

First, we tested whether consistent lags between pairs of neuronal firings (one neuron tends to fire earlier than the other during UP state) differed for CCGs with a trough compared with CCGs with a peak. When examining the difference in the timing of first spikes between pairs, 85 ± 18% of CCGs with trough and 91 ± 2% of CCGs with peak had differences significantly different from zero. For difference in mean spike, 51 ± 18% of CCGs with trough and 47 ± 14% of CCGs with peak had significantly nonzero means. Neither case for difference in first spikes or mean spikes are statistically different (*p* = 0.52 and *p* = 0.49). This indicated that consistent lags do not play a huge role in contributing antisynchrony. Indeed, consistent differences in spike timing would induce offset (nonzero lag) peaks, rather than troughs.

Next, Figure 6*C* shows that the average difference in mean spike time is greater in pairs of neurons with trough CCGs than peak CCGs (*p* = 1.6 × 10^−9^). This was not the case for difference of first spike times.

On the other hand, pairs with CCG troughs showed higher standard deviations in their neuron-to-neuron spike timing from UP state to UP state than pairs with CCG peaks. This was true when measured using both SD of difference of first spike time (*p* = 5.8 × 10^−6^) and SD of difference of mean spike time (*p* = 9.2 × 10^−13^). Thus, pairs of neurons with anticorrelations (CCG troughs) showed greater difference in spike time relative to each other and also more variable differences in spike time. This elevated variance in spike timing may be a means to create an anticorrelation rather than a shift or lag in the peak.

### Removal of burst spiking decreases anticorrelations between pairs of neurons in nonREM sleep

**Figure 7.**
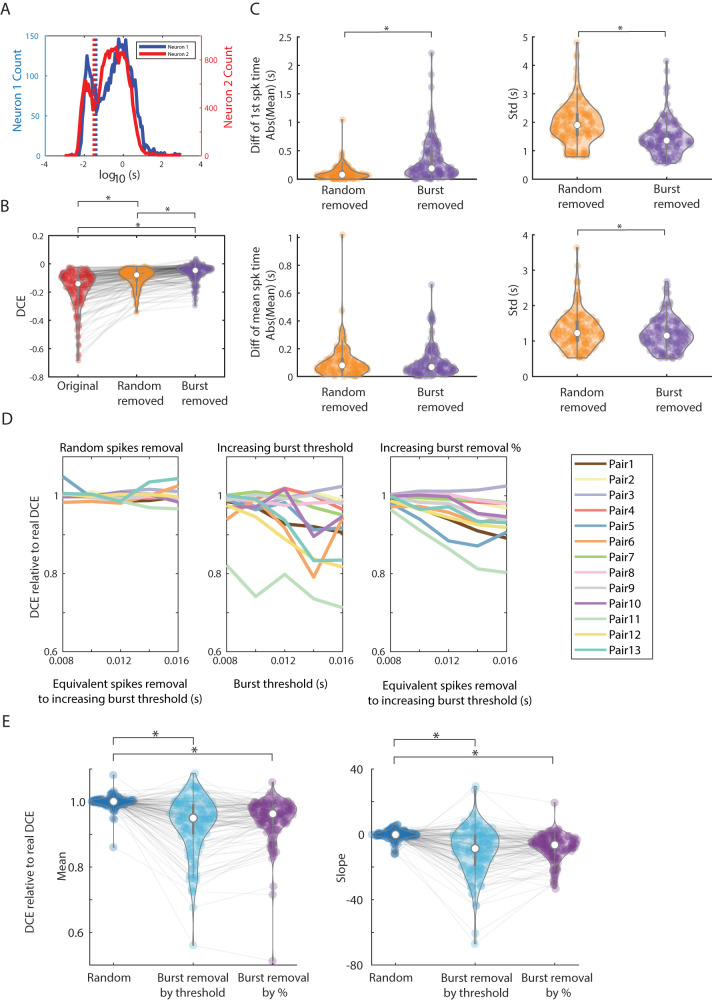
Removal of burst spikes decreases anticorrelations between pairs of neurons in nonREM sleep. ***A***, Examples of ISI histograms in log timescale from two single neurons. Many neurons display bimodal distribution in log timescale as shown here. We defined the burst spikes as the spikes with ISI less than the set threshold (see Materials and Methods). ***B***, DCEs are recalculated after removing burst spikes (right) or after removing the same amount of spikes in random fashion (middle). Only pairs with negative DCE originally are shown. Burst spike removal renders significantly lower amplitude anticorrelations (less negative DCE) than the original spike trains (*p* = 9.6 × 10^−10^) and random spikes removal (*p* = 5.6 × 10^−3^). Random removed pairs have significantly lower amplitude negative DCE than the original pairs (*p* = 9.6 × 10^−10^). ***C***, Temporal properties of spike trains resulting from burst removal versus random removal. We found that burst removal mainly affected difference of first spike time mean (*p* = 5.1 × 10^−12^) and SD (2.4 × 10^−10^), but not the difference of mean spike time (mean *p* = 0.62, SD *p* = 0.09). ***D***, We removed burst spikes in different ways to examine differential effects. Burst spikes were removed either by changing interspike interval used as the burst threshold (middle panel) or removing a numerically equivalent number of spikes but doing so by setting a percentage of burst spikes to remove in a random fashion equivalent the set threshold shown in Figure 6*A* (right panel). We progressively removed more spikes to uncover any progressive “dose effects.” The number of spikes removed in the left and right panel are equivalent to the number removed by moving the burst threshold (middle panel). DCE relative to real DCE (see Materials and Methods) is calculated so that the lower the value, the greater the effect of removing spikes on reducing the anticorrelation. The results from one of the representative datasets are shown here. ***E***, Collecting the mean and the slope of the DCE relative to real DCE from the full dataset, we found no significant difference between burst removal by threshold and burst removal by percentage (mean *p* = 0.1; slope *p* = 0.3). But they are both significantly lower than random spike removal. (random vs burst removal by threshold: mean *p* = 9.6 × 10^−10^; slope *p* = 9.6 × 10^−10^; random vs burst removal by percentage: mean *p* = 1.6 × 10^−9^; slope *p* = 2.1 × 10^−9^).

To further define UP state spiking dynamics contributing to anticorrelation, we looked to recent work (Levenstein et al., 2022) that demonstrated that many neurons showed bimodal ISI distributions (Fig. 7*A*). We used this bimodality to define a “burst” mode of firing: spikes with ISI in the lower of the two modes of firing of each neuron were defined as burst spikes (see Materials and Methods). We used this to determine whether “bursts” of high-frequency spikes might mediate anticorrelations. To analyze this, we found and removed burst spikes from UP state spike trains. As a control, we also removed the same amount of spikes in a random fashion. Figure 7*B* shows that anticorrelations of trough-containing CCGs are significantly reduced by removing bursts compared with both unperturbed spike trains (*p* = 9.6 × 10^−10^) and removing random spikes (*p* = 5.6 × 10^−3^). CCGs after random spike removal did show significantly higher DCE than the original pairs (*p* = 9.6 × 10^−10^).

To better study this, we quantified differences in mean spike times per cell per UP state (as in Fig. 6) after burst removal to determine if the burst removal effect may be mediated by spike timing changes. We found that removing bursts affected both the difference of first spike time metrics (paired *t* test: mean *p* = 1.8 × 10^−12^ and SD *p* = 1.6 × 10^−44^; Fig. 7*C*). This contribution of bursting to first-spiking rather than mean spiking may be consistent with higher average firing rates (more bursting) early in UP states. Furthermore, the difference of mean spike time SD was also lower when burst spikes were removed (*p* = 6.4 × 10^−10^).

Next, to further address whether consistent lags (one neuron fires consistently earlier than the other during UP state) between pairs affects anticorrelation, we calculated the DCE ratios for pairs that have zero means after random removal but nonzero means after burst removal. We also calculated the DCE ratios for pairs that have zero means after burst removal but nonzero means after random removal. None of the distributions of the ratios are significantly different for difference of first spikes (paired *t* test, *p* = 0.7) and mean spikes (*p* = 0.4). This again indicated that the consistent lags do not play a significant role in contributing antisynchrony.

We further removed burst spikes in different ways to examine the differential effects of those methods. In Figure 7*D*, the burst spikes are removed either by changing the ISI-based burst threshold for removal (middle panel) or removing a percentage of burst spikes in a random fashion within each UP state at the set threshold shown in Figure 7*A*, right panel (right panel; also see Materials and Methods). To make the analyses comparable, the number of spikes removed in the left and the right panel are equivalent to the number removed by moving the burst threshold (middle panel). DCE relative to real DCE (see Materials and Methods) is calculated so that the lower the value, the greater the effect of removing spikes on reducing the anticorrelation. As more burst spikes were removed, DCE relative to real DCE decreased (anticorrelation became weaker), while random removal of any spike did not have strong effects (Fig. 7*D*). In Figure 7*E*, we show that there is no significant difference between those different removal methods (mean *p* = 0.1; Slope *p* = 0.3), while they are both significantly different from random removal (random vs burst removal by threshold: mean *p* = 9.6 × 10^−10^; slope *p* = 9.6 × 10^−10^; random vs burst removal by percentage: mean *p* = 1.6 × 10^−9^; slope *p* = 2.1 × 10^−9^). This supports the notion that spike bursts affect the degree of anticorrelation but did not indicate a specific ISI cutoff of greater importance.

## Discussion

Here we report for the first time a nonREM sleep-induced anticorrelation, a novel finding that stands in contrast to the conceptualization of nonREM as a synchronizing state. We found that nonREM-specific anticorrelations are found in a clear minority of studied neuron pairs but occur in a manner not predicted by models using up-to-date knowledge of nonREM firing dynamics. These anticorrelations persist throughout 24 h periods consistently in pairs of neurons and so are nontransient. They are stronger when higher delta range power is seen in the LFP and less when gamma power is higher. Furthermore, they are able to be created without DOWN state firing and instead require only UP state firing to be created. Rather than being produced by generalized UP state dynamics within anticorrelated pairs, it is only the specific firing of pairs of neurons within individual simultaneous UP states that produces anticorrelation. More specifically, it is variable UP state-based spike timing between pairs of neurons that predicts anticorrelations—rather than systematic lags in firing. Finally, spiking occurring in bursts, at high frequencies, play a more important role than other spikes in creating anticorrelations.

Thus, this work contributes to phenomenon that has been underappreciated during nonREM which had been dubbed the “synchronized state.” We specifically assessed correlation structure at slower timescales (hundreds of milliseconds) rather than the well-known synaptic-timescale interactions below 5 ms lag (Fujisawa et al., 2008; English et al., 2017). This was due to our specific interest in delta wave-induced changes in synchrony at around the 1–4 Hz range (hundreds of milliseconds timescale). In our recordings, antisynchrony is found in nonREM in a manner not seen in REM or wake and by bursts of spiking occurring within single UP states. Other work has demonstrated anticorrelations either in models or anesthetized states (Renart et al., 2010), specifically in wake (Okun et al., 2019) or equally present in wake and nonREM (Euston et al., 2007; Gardner et al., 2019). Thus, what is novel here is the property of nonREM to not only synchronize some neuron pairs but to also actively desynchronize others.

While much prior work has focused on differences in firing properties of individual neurons during UP states (i.e., differential mean spike times relative to UP state start), few had postulated that those might lead to antisynchrony. UP state spiking has been postulated to play homeostatic roles (Levenstein et al., 2016; Watson et al., 2016; Liu and Watson, 2020) or memory consolidation roles in the brain (Bermudez Contreras et al., 2013; Liu and Watson, 2020).

A mixture of synchrony and antisynchrony in CCGs at a slow timescale similar to that reported here spiking was found in “grid-cells” in the entorhinal cortex that fired in spatially modulated patterns (Gardner et al., 2019). However, in the entorhinal cortex, that work did not demonstrate a strengthening of anticorrelation in nonREM because those anticorrelations were already strong in wake. Other work in the prefrontal cortex showed that slow timescale correlations (multisecond) and anticorrelations during wake maze running were found at faster timescales (subsecond) in nonREM sleep (Euston et al., 2007). Thus, any synchrony effects of nonREM were assumed to be positive or neutral in influence relative to wake/REM states, rather than the sometimes-negative effects shown in these experiments. Of note, all of our recordings were in the homecage and so may not have engaged some correlation patterns drive-able by more active behavior as used in these papers. Future work could determine whether the neuron pairs driven to greater antisynchrony in nonREM are those also driven by active waking engagement. However, overall synchrony during the delta-rich nonREM state, which we also observe, has been assumed to be pervasive, leaving induction of novel anticorrelations less explored.

Using a variety of methods, we found that delta power positively modulates trough depth within nonREM sleep. This was based on analyzing CCGs in nonREM epochs with different spectral properties. One question that arises is what governs nonREM epochs to have differential delta, gamma, or other frequency band powers. We found some explanations for this including that longer duration nonREM epochs show more delta and less gamma power. However, what governs this duration remains unknown. One leading factor could be circadian time, but this dataset is not able to analyze that for a variety of reasons and future work will need to follow up on the modulators of duration of nonREM epochs.

Mechanistically, we found that UP state spikes alone are sufficient to induce this anticorrelation (Fig. 5), found by excluding spikes except those detected explicitly in UP states. Therefore, this anticorrelation does not require for example DOWN state firing cells recently reported (Todorova and Zugaro, 2019). Furthermore, the generalized per-cell UP state firing tendencies were not sufficient to induce anticorrelations, since shuffling spike trains across UP states did not show anticorrelation; only two-neuron spike trains simultaneously recorded from the same UP state incidents yielded anticorrelation.

Additionally, removing burst spikes reduced anticorrelations. Removing these spikes mainly affected the first spike timings (Fig. 7*C*), while in our data mean spike timing are also significantly different between CCGs with troughs and peaks (Fig. 6*C*). Interestingly, removing burst spikes decreases antisynchrony and increases the absolute value of mean of the difference of first spike time, while CCGs with a peak had lower mean of the difference of mean spike time compared with CCGs with a trough. However, the trends of standard deviations in both cases are both higher for more antisynchronized pairs implying that spike timing variance may play a role.

One interpretation of our findings is that there could be microstructures in the cortex, with different subnetworks running at a different timing to increase the signal-to-noise ratio and modulate linkages and disconnections between subnetworks because of Hebbian learning. This is supported by the phenomenon we observed that the synchrony and antisynchrony effect is higher during nonREM (Fig. 3), which is when the memories are known to be processed and solidified (Rasch and Born, 2013). Another supporting point is that removing burst spikes have a significant effect on reducing antisynchrony, and the burst spikes are suggested to encode useful information during this “activated state” (Levenstein et al., 2022). Based on this, it would be important to determine whether learning does affect the CCG structures at a slow timescale. Finally, modeling work suggests desynchronization of excitatory versus inhibitory populations could also induce such observed antisynchrony (Renart et al., 2010). Unfortunately, our analyses have been unable to provide definitive proof for this hypothesis, and further mechanistic work is required to understand our findings.

We see this work as opening a new door to understanding the full breadth of the effects of nonREM sleep on cortical firing. Further investigations into both the causes and outcomes of this induced anticorrelation during the synchronized state can lead to better understanding of the role of spiking in nonREM.

## Synthesis

Reviewing Editor: Michaël Zugaro, CNRS, Collège de France, Inserm Decisions are customarily a result of the Reviewing Editor and the peer reviewers coming together and discussing their recommendations until a consensus is reached. When revisions are invited, a fact-based synthesis statement explaining their decision and outlining what is needed to prepare a revision will be listed below. The following reviewer(s) agreed to reveal their identity: Michael Okun, Ralitsa Todorova.

The manuscript describes anti-correlations in cortical neuronal data during UP states of slow wave sleep. The major claims are that (1) anti-correlations are not expected in nonREM sleep and do not simply follow from each cell's dynamics relative to the alternating UP and DOWN states, (2) both the synchronous and anti-synchronous relationships between pairs of cells are more pronounced in nonREM than other states, (3) these relationships covary with specific LFP frequencies, (4) they reflect correlations within an UP state and (5) in particular bursting activity.

The question is interesting but the Reviewers have raised a number of major concerns regarding the described research, and have made both general and more specific suggestions to strengthen the study.

1. General Suggestions

The main issue is that it is actually not clear that the key finding of the work is new. While the authors base their expected distribution of correlations during UP states on Luczak et al. (PNAS 2007), there are two related and highly relevant studies from the very same lab that were not referenced or discussed.

The first one is Renart et al. (Science 2010), in which one of the key points is that in the asynchronous state (and in particular UP states when considered in isolation) the distribution of pairwise correlations is much broader than one might expect, in particular leading to many neuronal pairs with a negative correlation (see Fig. 4 &Fig. S10 there). The theoretical mechanism which explains this is the major contribution of that paper. Therefore the observations reported here seem to be precisely what one would expect to see based on that work. This study is based on modeling and data from anesthetized animals. While this remained to be confirmed in freely sleeping animals (as in the current work), the negative correlation was therefore not really unexpected.

The second publication is Okun et al. (Nature 2015), which classified neurons by their coupling to population rate on timescales of 100-300 ms. Some neurons were found to have negative coupling. Any such neuron is expected to have a negative correlation with any neuron with positive coupling. That work includes a relatively straightforward population coupling model which predicts pairwise correlations (including negative correlations). Yet, this work was done in animals engaged in visual tasks, so this does leave room for similar investigations specifically during slow wave sleep.

Other studies that have shown negative CCGs, in particular in sleep, include Euston2007 (Figure 1A Cell pair 3), Gardner2019 (Figure 1a), and Fujisawa2008 (Figure 3a).

Therefore, the general suggestions are to recenter the manuscript on novel findings (reevaluating novelty based on the above studies), reformulate the logic of the study accordingly, and perform additional analyses to deepen and strengthen the new claims.

2. More Specific Suggestions

In the first place, negative cross-correlograms can be considered non-trivial only for excitatory neurons. Cross-correlograms with negative troughs are clearly expected for inhibitory connections. Yet, this does not seem to have been addressed in the current manuscript. How are the anti-synchronous CCGs described here different from classic negative CCG troughs due to mono- or poly- synaptic inhibition? If the anti-correlations do merely reflect inhibitory connections, this needs to be made clear.

In the discussion, the authors suggest the anti-synchrony may be due to disconnected sub-networks, which is an intriguing possibility alternative to the somewhat trivial explanation of an inhibitory connection between a pair of neurons. The manuscript would be considerably stronger if it contained some support of this alternative interpretation. The authors could show examples of two such sub-networks: the CCGs of 4 cells, where A and B (of one putative sub-network) are correlated with each other and anti-correlated with the other two cells C and D (of an opposing putative sub-network), and vice versa. Such examples would support the exciting subnetwork theory while also preventing potential readers from writing off the findings as trivial consequences of inhibitory connections.

Throughout the manuscript, the authors compare metrics of CCGs computed in different states (or in different time windows in the analysis of hour-long epochs), including DCE amplitude and percent significant DCEs. A major concern is that a CCG containing more spikes would have higher statistical power than a CCG containing fewer spikes, which would bias all comparisons in the favor of the CCG with higher sampling. Especially in the case of anti-correlations, it is much easier to see silence when one has sufficient sampling and the baseline spike counts are high. In the extreme case, when a CCG has very few spikes (e.g. due to data restricted over a short period such as REM epochs), the baseline spike counts could be so few that one may not be able to tell the anti-correlation silence from the baseline. This would result in a DCE value of a lower amplitude and a non-significant t-test (of the CCG baseline vs the CCG middle comparison). To eliminate this statistical power issue, it would be necessary to include a downsampling control. In such a control, when comparing any two CCGs, then the CCG containing more spikes would be downsampled to match the number of spikes in the other CCG, ensuring that statistical power is equivalent in both cases. Downsampling would be a fair way to convince readers that the results in Figure 2 (especially 2C), Figure 3, and Supplementary Figure 2 are qualitative and not due to fluctuations in statistical power or better sampling in nonREM epochs.

A potential major issue refers to Figure 2A (and B) which shows all the CCGs that have passed a criterion. The goal of this section is to convince the reader that CCG relationships are stronger in nonREM sleep than in Wake and REM sleep. I am concerned about the selection procedure here: "The stacked CCGs are sorted from deepest trough to highest peak based on CCGs at nonREM state. Only CCGs with a significant peak or trough are shown." It is not clear if the "CCGs with a significant peak or trough" refers to the pairs that yielded a significant peak/through in ANY brain state or specifically the pairs that are significant in nonREM - hopefully, the former (in which case, this should simply be clarified in the text), as the latter would be a critical statistical pre-selection issue, as it would be misleading to compare (in a statistical test or in a visual representation) pre-selected values to other (non-selected) values.

3. Minor Points

There is a bit of confusion with some of the descriptions of the time scale of the studied phenomenon. On multiple occasions, the time scale of the correlations studied here is described as "slow". The abstract and introduction, including the references to "slow" time scales and "time scale of seconds", leave the false impression that the manuscript describes pairs of cells that fire in different UP states. After going through the methods, it seems that the correlations described here are those on a time scale under 125 ms. This is because slow relationships (coarser than 500ms as stated in the methods) are removed during the normalization step, and the troughs/peaks are quantified as the mean values within the [-125 +125] ms window in the normalized CCG after canceling out relationships at +/-[750 1000] ms. If this understanding is correct, then the analyses describe pairs of spikes within 125 ms of each other, much faster than the time scale of "seconds" mentioned in the abstract and the discussion. The discussion even mentions definitions of "slow" and "fast" (when referring to another article) as "multi-second" and "sub-second", adding to the confusion as in such a framework the current correlations should certainly be considered "fast" (indeed, the time scale of the CCG troughs in the cited article Euston2007 are not any faster than the ones described here). "Slow" and "fast" need to be defined early on and used consistently throughout the manuscript.

The authors should be commended on their simulation analyses in Figure 1. It is a pity that a single example is shown in Figure 1Cii to represent all this work. The authors would be well advised to include the stacked CCGs that the simulation produced. This would allow the reader to better compare the differences between the simulation and the original data with network interactions intact.

In general in all panels with stacked CCG, the data is selected to include only pairs with significant peaks and troughs. While this showcases the most interesting parts of the stacked CCG (keeping in mind that 10% of all CCG pairs are significant), the manuscript would be more complete if the pooled stacked CCGs were displayed in their entirety, perhaps in addition to these zoomed-in stacked CCGs of significant pairs only.

It is not clear why Figure 2A shows the stacked CCGs of a single recording session rather than the pooled data. It is interesting to show representative examples, but only if the complete data is also shown somewhere in the manuscript, and it is missing here. In the same figure, one wonders why panel B displays data from 10 pairs only, again not showing the data.

In the accompanying text, the authors include many comparisons of different pre-selected sets of CCG pairs, referring to "nonzero DCE pairs". Does "nonzero" here mean "significant"? Are all DCE values in CCGs with a nonsignificant t-test set to zero? This should be clarified.

It is puzzling that the authors compared the DCE amplitudes of many sets of pre-selected pairs, including: pairs significant in nonREM sleep, pairs significant in Wake, pairs significant in REM sleep, and pairs significant in all those states (assuming this means the pair needs to be significant in all three states rather than a single one of them, although the n-s are not reported). However, the DCE amplitude is not independent from the significance test, which puts the validity of first three of those sets of tests into question. The authors should remove such biased comparisons and only keep comparisons which treat all brain states equally (with downsampling as described above). It is also puzzling why the authors did not include the simplest of all comparisons: comparing amplitudes of all DCEs without any pre-selection.

The methods regarding the LFP analysis could be clearer: given that the central query of the manuscript concerns nonREM sleep, one assumes that data from other brain states were excluded here. But then how should one interpret the findings described here? Recordings were scored for delta and gamma power divided into bins of one hour, which seems quite long. A single nonREM cycle can be expected to last less than an hour, and delta and especially gamma rhythms are much more transient. It would be helpful if the authors could comment on how they interpret gamma-rich hours. When would such epochs be expected to take place? Again, downsampling for this analysis as well as the analysis in Supplementary Figure 2 would help ensure this is not a samplig issue (e.g. due to less sleep data being present in a given hour).

The shift analysis in Figure 4 is very convincing that the anti-correlations are due to network interactions within a single UP state. It is a bit surprising that all of the pairs shown seem to be highly positively correlated (one would have expected about a quarter of the pairs to show a convincing peak based on the simulation results in the very first section which also erase the fine-timescale relationships). Why are the values on the sides of the CCGs (around 1.5s) so high in some cases? It would also be interesting to see entire set of stacked CCGs (rather than pre-selected CCGs with significant peaks/troughs) here as well as Figure 1 for curious readers.

Data from 13 pairs are shown in Figure 6D. The authors should represent the whole data set with a curve showing the mean +/- s.e.m.

The legend in Supplementary Figure 2D could be clearer. The x-axis starts at 0.4: were there no Pearson's R lower than 0.4?

Incidentally, it would generally help if the authors used analyses and nomenclature that is already well established, instead of coming up with new ones. For example, DCE seems to be what is commonly known as jitter-corrected CCG (e.g. see Smith and Kohn, JNeurosci. 2008 or Renart et al., Science 2010).

An additional example is the rather convoluted analysis of Fig. 4 to investigate the contribution of specific populations of neurons that do spike in DOWN states. A simple, logical and straightforward approach would be to find neurons that spike during DOWN states and analyze them separately.

In this respect, it is also worth noting that in addition to Valero et al., the work of Zucca et al. (Current Biology 2019) reported that PV cells can spike during DOWN states.

4. Additional Points

The point of Figure 3 was not entirely clear. The illustrated effect appears to be the direct byproduct of what we see in Figure 2: hours in which the rats slept (mostly nonREM with high delta power and low gamma) had stronger CCGs than hours in which the animals were awake (with low delta and high gamma).

Table 1. # of pairs for some reason is 2-fold higher than possible. For example, a recording with 101 neurons can only have 101*100/2 = 5050 pairs ("nchoosek" where n=101 and k=2).

p2 "details of that spiking across multiple neurons is believed to dictate" ---> details of spiking across multiple neurons are believed to dictate

p3 The works of Steriade lab are usually credited for intracellular characterization of UP &DOWN states.

p7 "For examples" ---> For example

p9 "CCGs resulting after random removal" ---> CCGs after random spike removal
