# Supplementary figures and images for: Desynchronization Increased in the Synchronized State: Subsets of Neocortical Neurons Become Strongly Anticorrelated during NonREM Sleep
Source: eNeuro. 2025 Mar 11;12(3):ENEURO.0494-22.2025. doi: 10.1523/ENEURO.0494-22.2025 (PMC11934222; doi:10.1523/ENEURO.0494-22.2025)

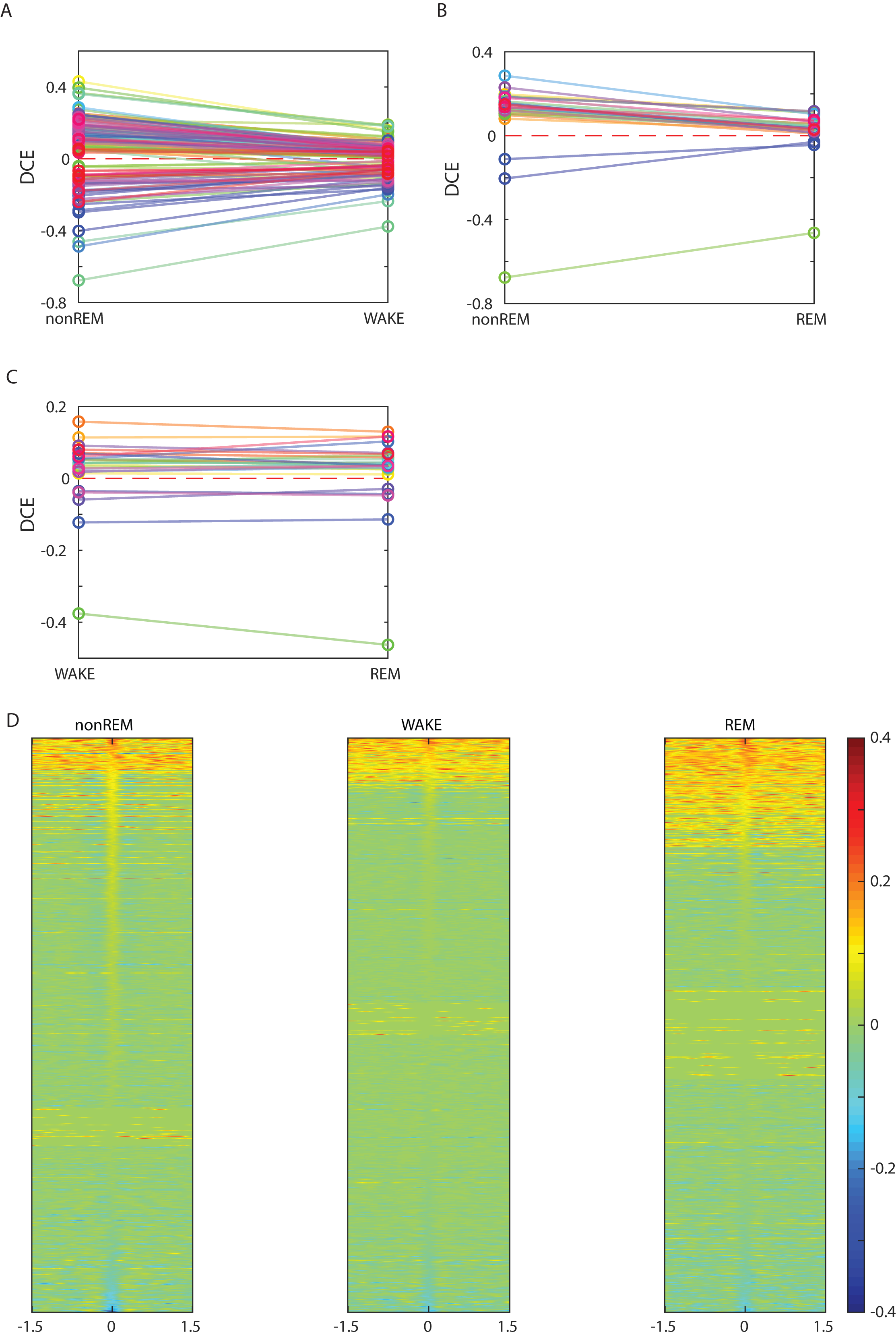

Supplement: Figure 3-1 — CCG Pairwise comparison between sleep states from all recordings.(A) Pairwise comparison between nonREM and wake on all the pairs in the dataset that have nonzero values in these two states (P = 2.58 × 10^−36^).(B) Pairwise comparison between nonREM and REM on all the pairs in the dataset that have nonzero values in these two states (P = 2.01 × 10^−14^).(C) Pairwise comparison between wake and REM on all the pairs in the dataset that have nonzero values in these two states (P = 0.5).(D) Stacked CCGs from full dataset. Each stack is sorted by its own middle amplitudes. Download Figure 3-1, TIF file. [file eneuro-12-ENEURO.0494-22.2025-s002.tif]

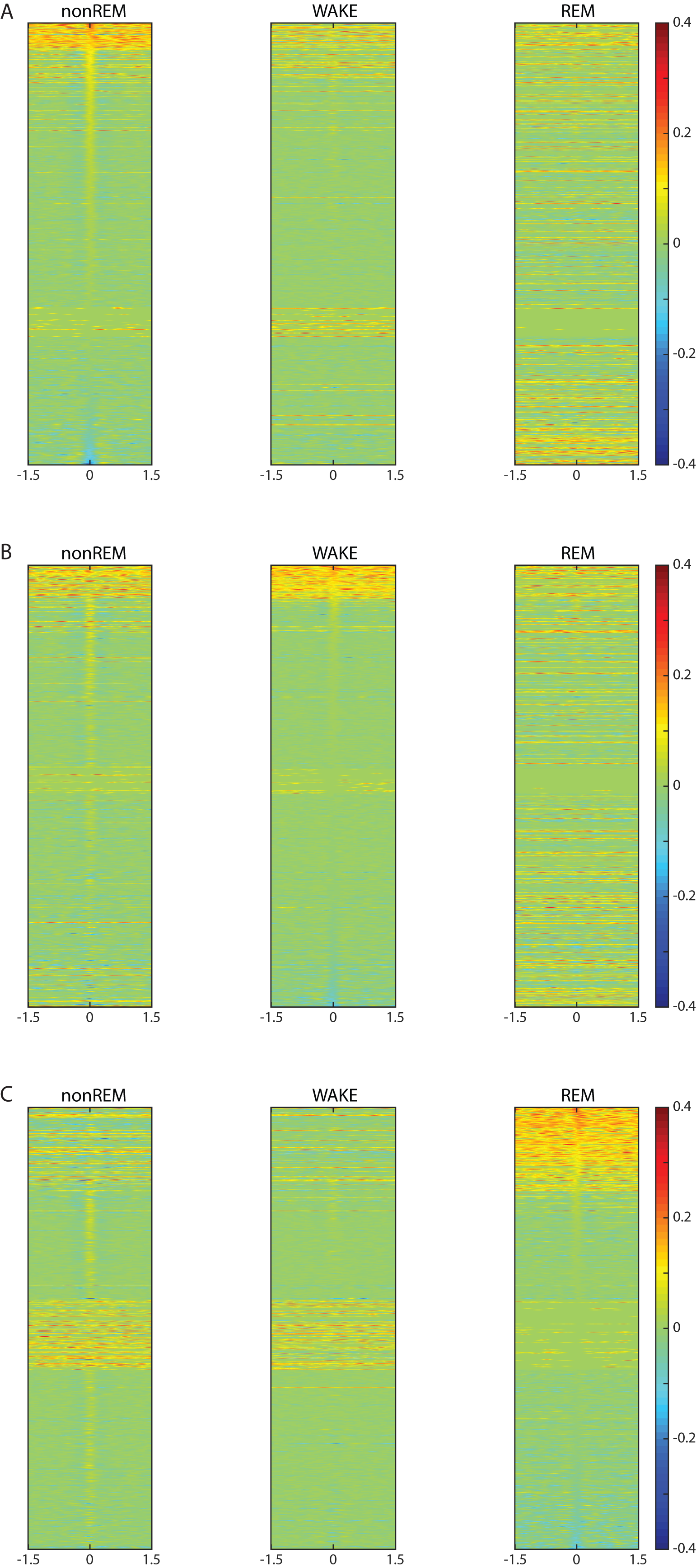

Supplement: Figure 3-2 — Stacked CCGs between sleep states from all recordings sorted by different sleep states.(A) All the Stacked CCGs are sorted by nonREM.(B) All the Stacked CCGs are sorted by wake.(C) All the Stacked CCGs are sorted by REM. Download Figure 3-2, TIF file. [file eneuro-12-ENEURO.0494-22.2025-s003.tif]

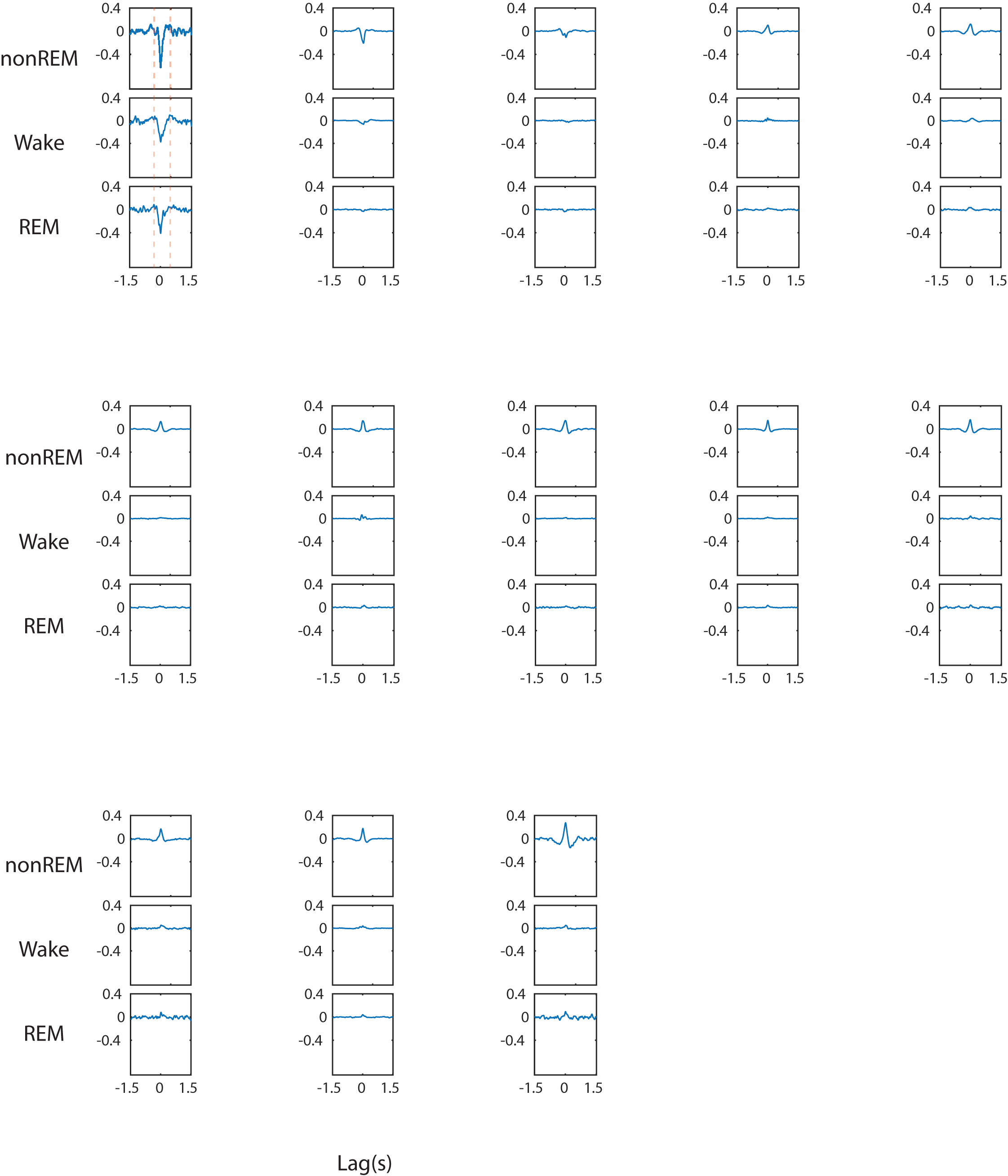

Supplement: Figure 3-3 — Comparison of CCG samples between states in one recording. CCGs with their DCEs shown in Figure 3B are displayed here. The CCGs are sorted by the DCEs in nonREM. Each column of three CCGs is from one neuron pair. The red dot-lines in the top left panels denote the width of trough in Wake. CCGs were Gaussian smoothed at width 10 ms. Download Figure 3-3, TIF file. [file eneuro-12-ENEURO.0494-22.2025-s004.tif]

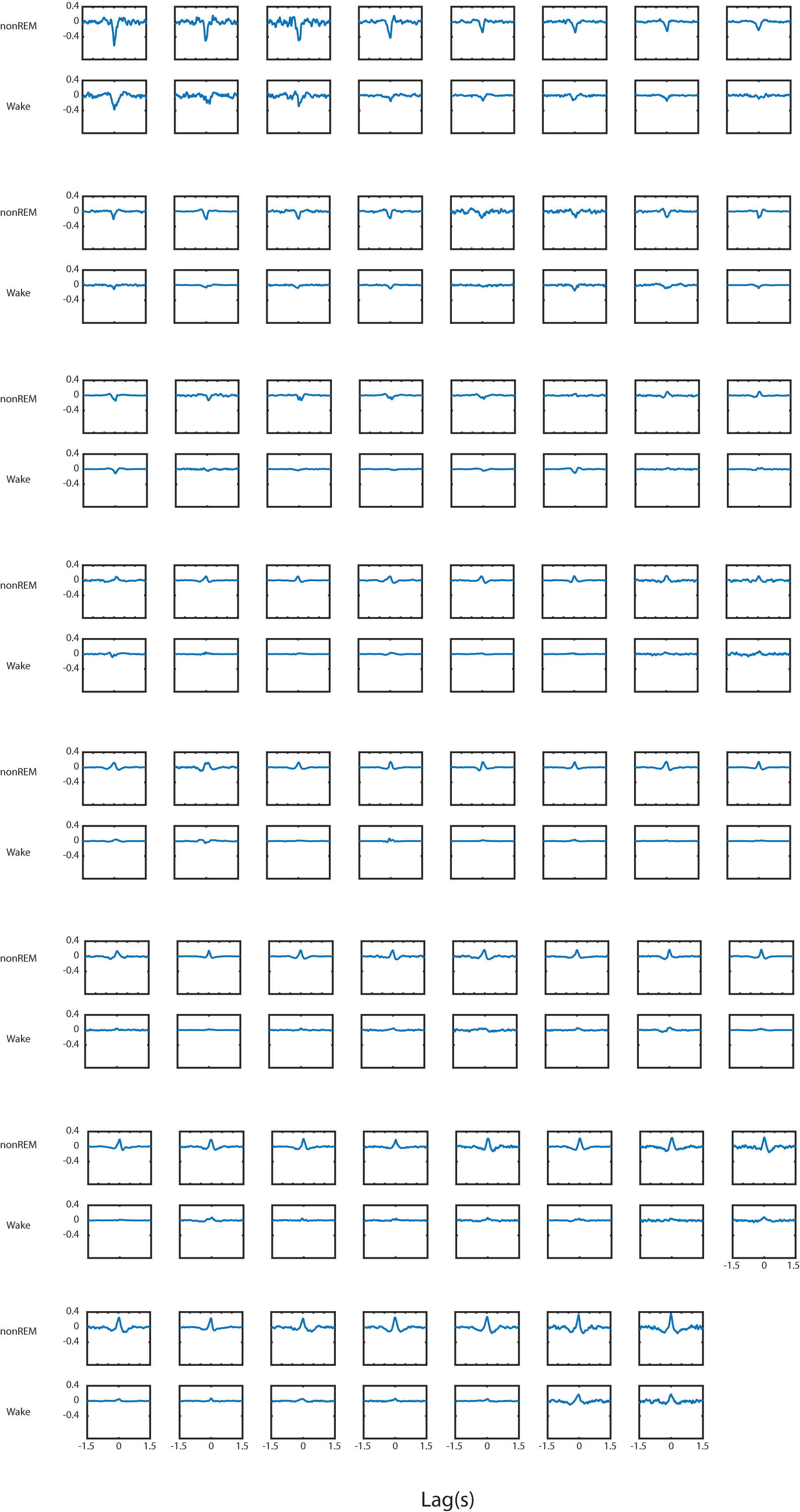

Supplement: Figure 3-4 — Comparison of CCG between nonREM and Wake in example pairs. All the pairs with nonzero DCE in both nonREM and Wake in one recording are compared. Each column of two CCGs is from one neuron pair. The CCGs are sorted by the DCEs in nonREM. CCGs were Gaussian smoothed at width 10 ms. Download Figure 3-4, TIF file. [file eneuro-12-ENEURO.0494-22.2025-s005.tif]

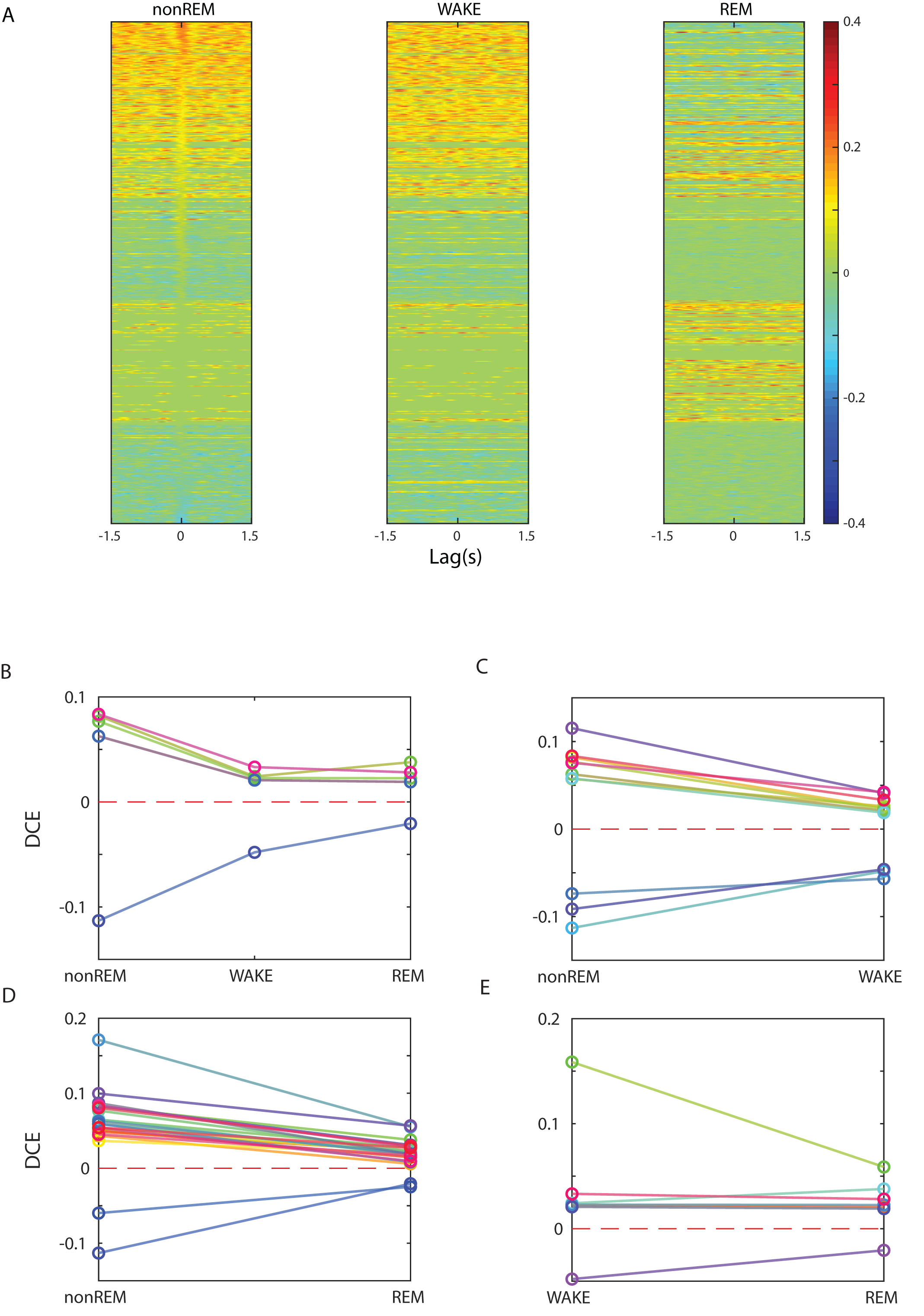

Supplement: Figure 3-5 — Downsampled CCG Pairwise comparison between sleep states from all recordings.(A) Stacked and downsampled CCGs across sleep/wake states from full dataset to demonstrate the range of correlation structures. Each stack is sorted by nonREM CCGs’ middle amplitudes. All CCGs are shown including zero and nonzero DCE.(B) Comparison between nonREM, wake, and REM on all the pairs in the dataset that have nonzero values in all three states (One-way ANOVA P = 3.17 × 10^−11^; nonREM VS wake P = 1.83 × 10^−9^; nonREM VS REM P = 1.13 × 10^−9^; REM VS wake P = 0.74).(C) Pairwise comparison between nonREM and wake in all the pairs in the dataset that have nonzero values in these two states (P = 6.25 × 10^−12^).(D) Pairwise comparison between nonREM and REM in all the pairs in the dataset that have nonzero values in these two states (P = 7.31 × 10^−19^).(E) Pairwise comparison between wake and REM in all the pairs in the dataset that have nonzero values in these two states (P = 0.1). Download Figure 3-5, TIF file. [file eneuro-12-ENEURO.0494-22.2025-s006.tif]

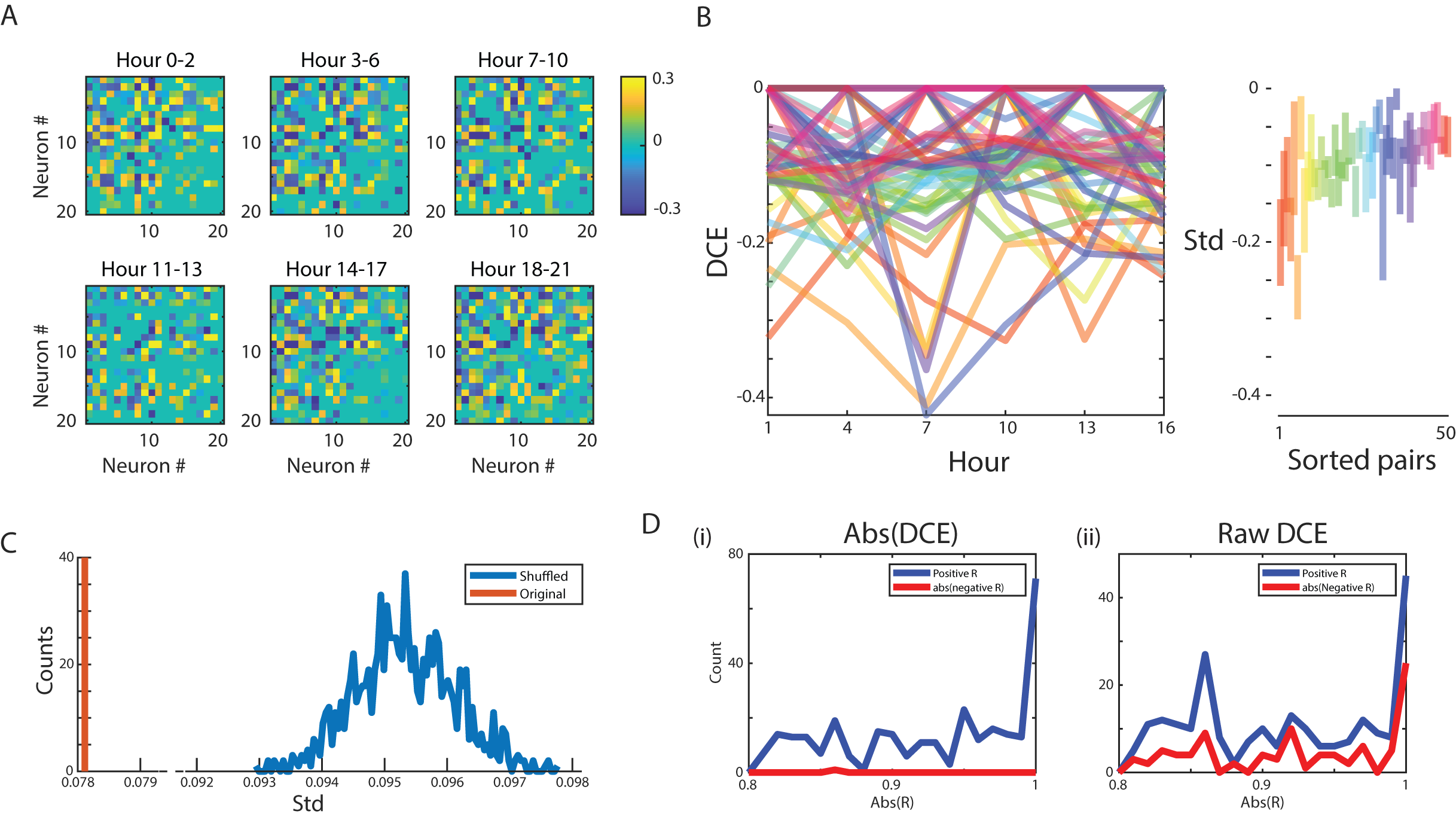

Supplement: Figure 4-1 — Strengths of pairwise correlations across the population are coordinated over time.(A) Recordings are divided into epochs of three hours. The twenty neurons that are the least synchronized with other neurons (neurons that have the most negative DCE counts during nonREM) are selected for this display. Their DCEs during nonREM at those three-hour epochs are then calculated and shown as the matrix elements. Color scale shows DCE value with each value in the matrix representing the DCE of one neuron pair. Note overall similarity of DCE matrix over multiple hours of recording.(B) At left, time courses of DCE for fifty example pairs with the most negative DCEs during nonREM. At right, standard deviations respectively were calculated for each pair and were shown sorted by its DCE value during nonREM epoch.(C) Comparison between the mean std from the original matrix and matrices where values within each three hours are shuffled (Z-test, P = 1.4 × 10−168). The std of the real data is much less than the shuffled, showing the conservation of the correlation structure over time.(D) Correlation between pairs over time. As one pair of neurons becomes more strongly correlated, do others at the same time? The amplitude of DCE between pairs tends to be positively correlated from one timepoint to the next.(i) The absolute values of the time courses of DCE for the twenty neurons’ pairs are first taken. The Pearson correlations between the resultant time courses for each pair are then calculated. Only significant correlations are counted (P < 0.05). The absolute values of the correlation coefficient R are taken and the distribution of the negative Rs and positive Rs are shown. Generally, we see many more counts of significant positive correlations than negative correlations.(ii) Similar approach to (i), but without taking the absolute values of the time courses of DCE. Note the lower amplitude red line in (i), showing that most of the negative Rs are contributed by larger positive DCEs [file eneuro-12-ENEURO.0494-22.2025-s007.tif]

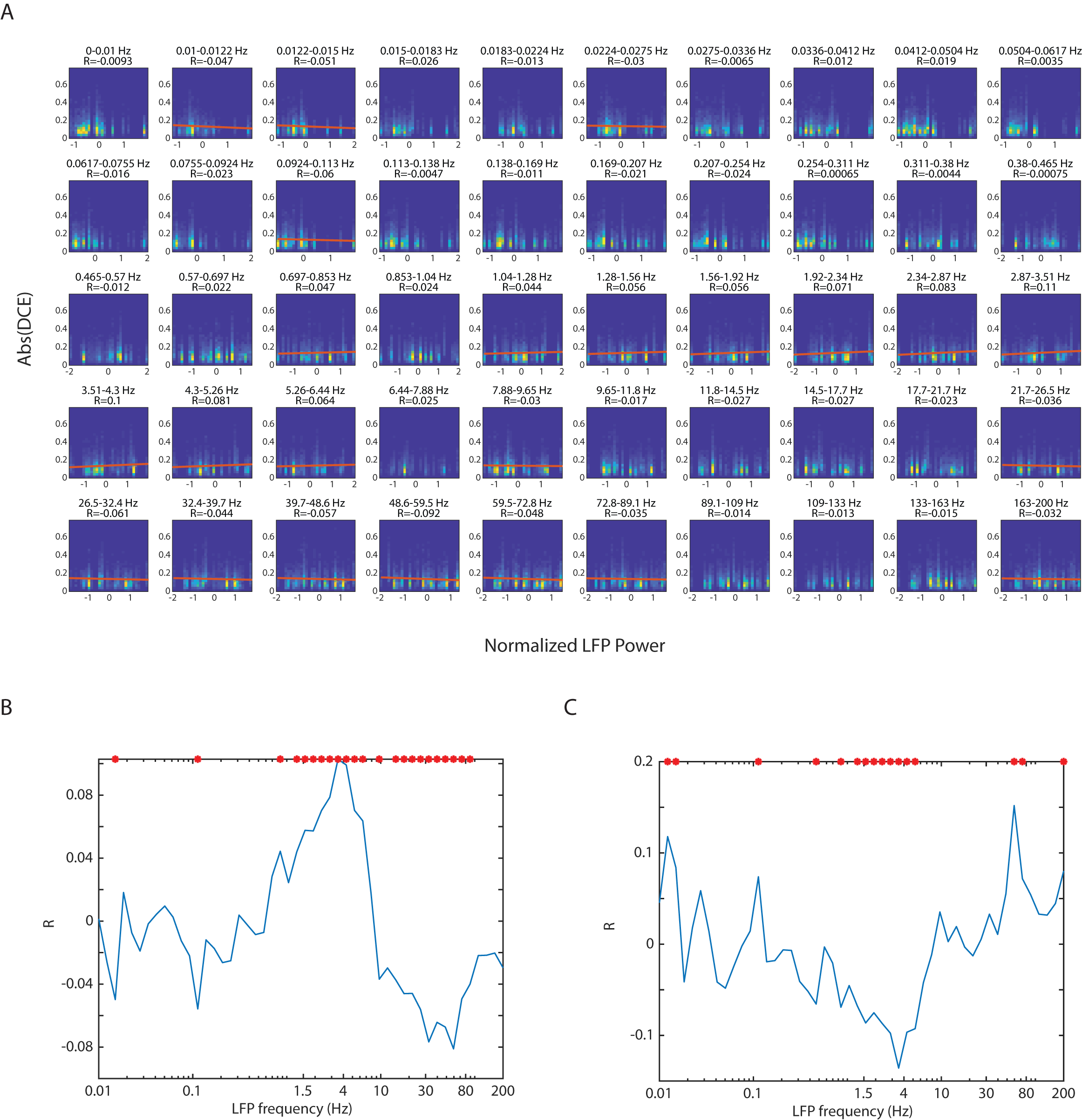

Supplement: Figure 4-2 — Full data for Figure 4. Separating three-hour epochs with positive DCE from negative DCE and analyze them independently yields consistent result(A) Density plots of mean LFP power versus the absolute values of DCE in each frequency band shown in Figure 4. For density plots with statistically significant R (P < 0.05), the best linear fits are drawn as red lines.(B) Grouping three-hour epochs with positive DCE and find the R values of mean LFP power versus DCE for each of many frequency bands. Relation between the R values and frequency bands are shown. Significant R values are denoted with red asterisks (P < 0.05).(C) Similar approach to (B) but for epochs with negative DCE. Download Figure 4-2, TIF file. [file eneuro-12-ENEURO.0494-22.2025-s008.tif]

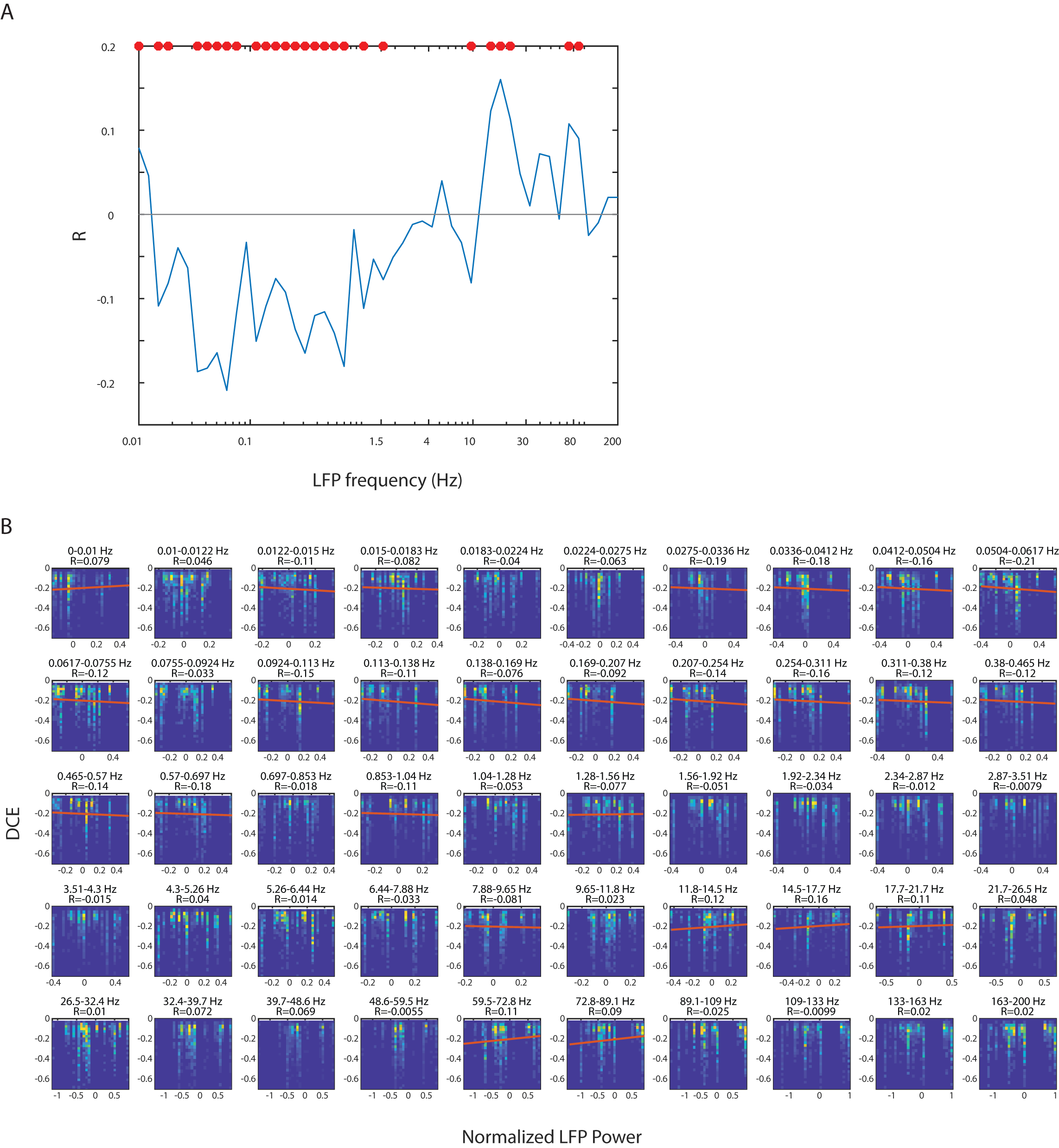

Supplement: Figure 4-3 — Alternative analysis to verify the correlation between band power and DCE in nonREM states.(A) Pearson Correlation between the LFP power and the DCEs calculated in an alternative way. Given a frequency band, each nonREM epoch is ranked into one of four power quartiles and the CCGs are generated for each pair. Correlation between the power and the DCEs calculated from the CCGs in that frequency band can then be calculated. Significant R values are denoted with red asterisks (P < 0.05). Qualitatively similar overall findings to figure 4B, despite different methodology.(B) Raw data underlying plot in (A). For density plots with statistically significant R (P < 0.05), the best linear fits are drawn in red lines. Download Figure 4-3, TIF file. [file eneuro-12-ENEURO.0494-22.2025-s009.tif]

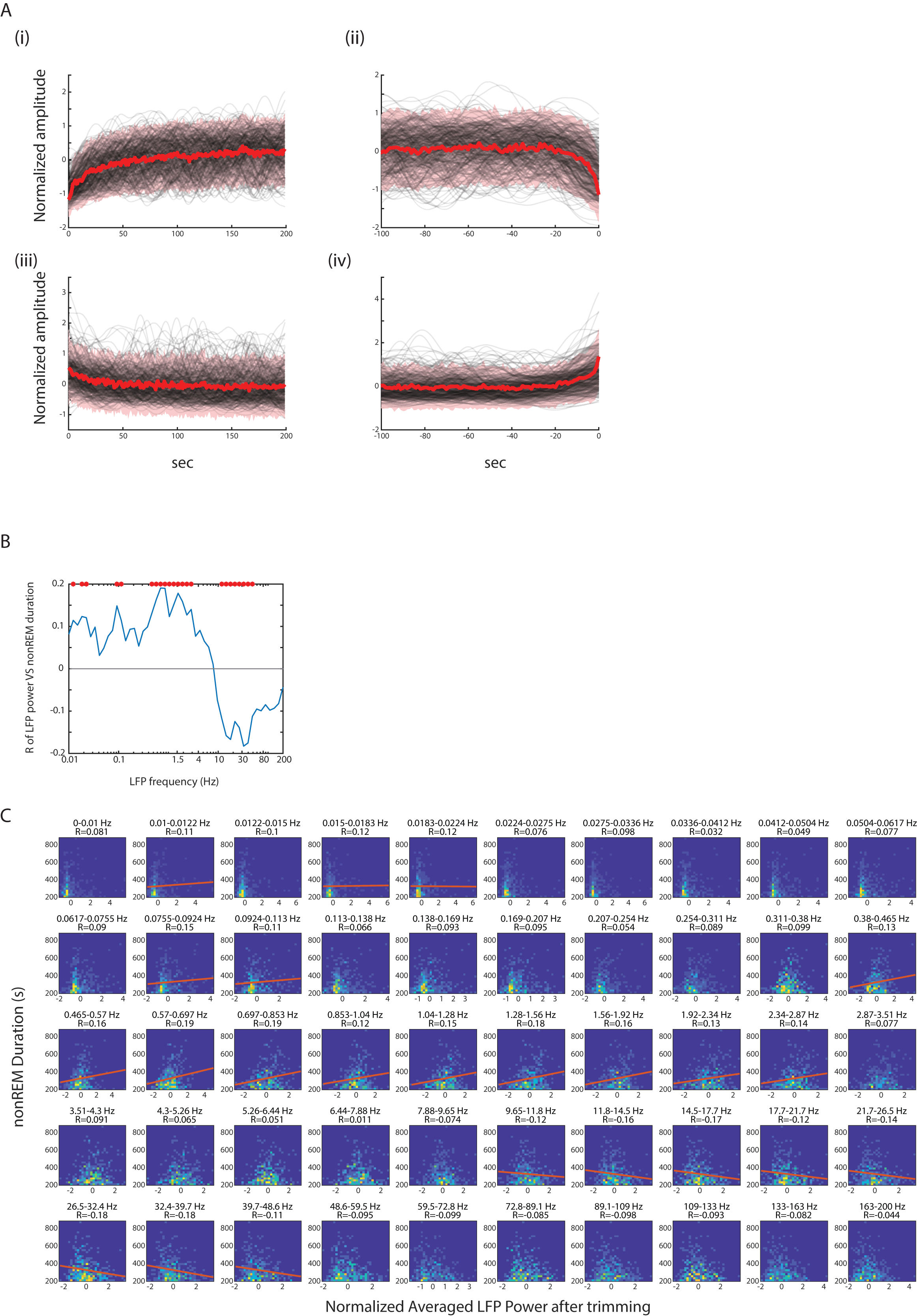

Supplement: Figure 4-4 — NonREM epoch duration is positively correlated with delta power and negatively correlated with gamma power.(A) We initially found duration of epoch correlates with delta power and negatively correlates with gamma power (similar to 4-4B, but not shown), but to prevent contamination and overly strong findings by ramp-up and ramp-down dynamics, we analyzed only plateaus of each epoch using the following analysis. We found putative contamination of findings by the fact that regardless of epoch duration,timecourse of delta power (i) in the first 200 secs and (ii) in the last 100secs of each nonREM epoch (>200 secs) did not vary. This leads to short duration epochs having low delta and high gamma values not representative of their plateau value, potentially falsely over-driving correlations. Gamma powers are shown in (iii) and (iv). Each power trace is shown as a grey line. Mean values are shown in red lines and standard deviation are denoted by light red area.(B) After correction: pearson correlation between the duration of nonREM epochs (>200 secs) and the mean LFP power in each frequency band. Band power in the first 120 secs and last 50 secs are removed when averaging. Significant R values are denoted with red asterisks (P < 0.05).(C) Raw data underlying plot in (B). For density plots with statistically significant R (P < 0.05), the best linear fits are drawn in red lines. Download Figure 4-4, TIF file. [file eneuro-12-ENEURO.0494-22.2025-s010.tif]

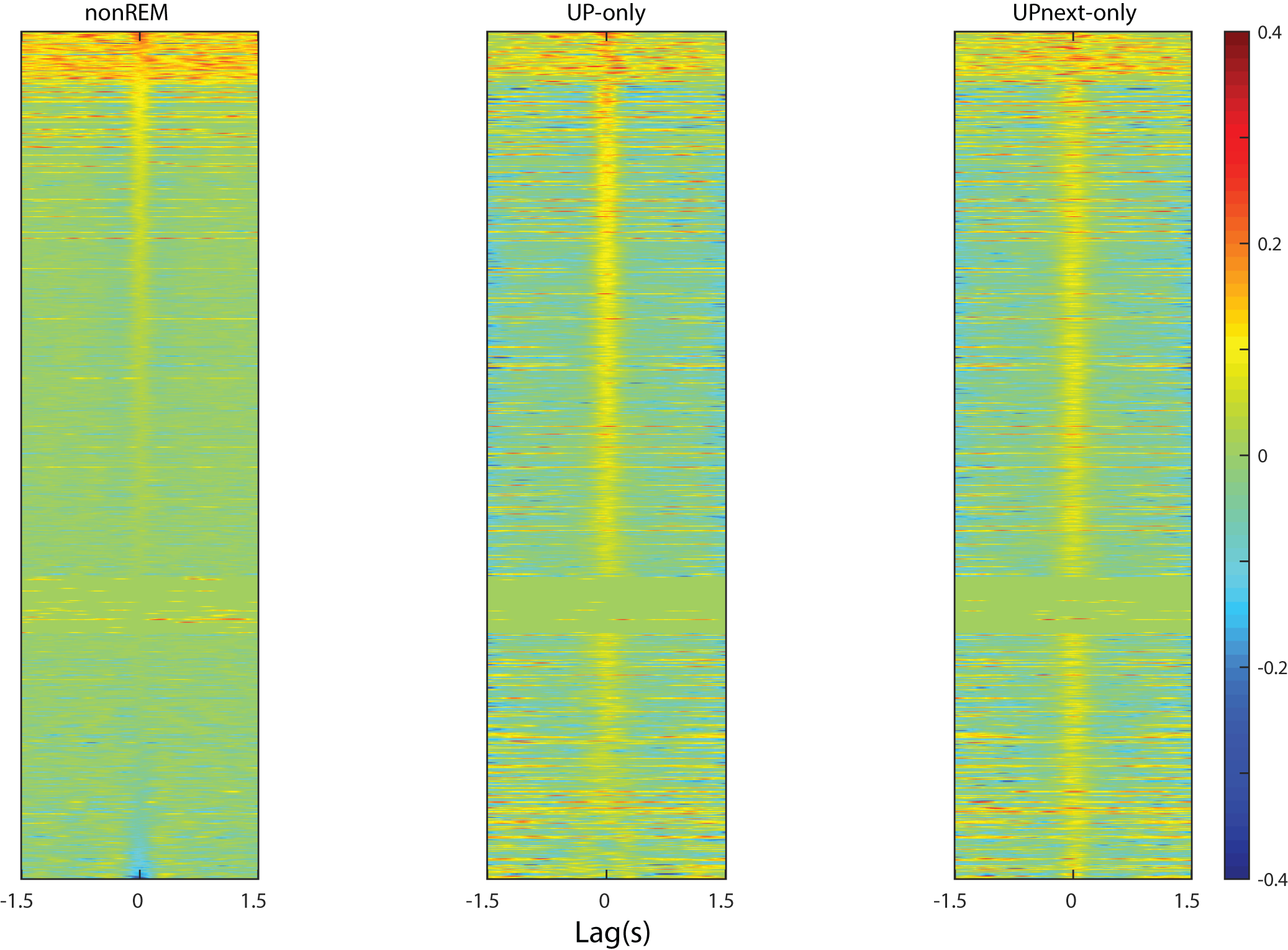

Supplement: Figure 5-1 — Complete comparison of the stacked CCGs in nonREM, UP-only, and “UPnext-only” states from all dataset (not restricted to significant pairs). Each stack is sorted by nonREM CCGs’ middle amplitudes. All CCGs are shown including zero and nonzero DCE. Results are consistent with significant-only pairs. Download Figure 5-1, TIF file. [file eneuro-12-ENEURO.0494-22.2025-s011.tif]
